# Functional network of contextual and temporal memory has increased amygdala centrality and connectivity with the retrosplenial cortex, thalamus, and hippocampus

**DOI:** 10.1038/s41598-023-39946-1

**Published:** 2023-08-11

**Authors:** Thays Brenner Santos, Juliana Carlota Kramer-Soares, Cesar Augusto de Oliveira Coelho, Maria Gabriela Menezes Oliveira

**Affiliations:** 1 Departamento de Psicobiologia, Universidade Federal de São Paulo - UNIFESP, São Paulo, 04023-062 Brazil; 2 Neuroscience and Mental Health, Hospital for Sick Children, Toronto, ON M5G 0A4 Canada; 3 Universidade Cruzeiro do Sul - UNICSUL, São Paulo, 08060-070 Brazil

**Keywords:** Neuroscience, Cognitive neuroscience, Emotion, Learning and memory

## Abstract

In fear conditioning with time intervals between the conditioned (CS) and unconditioned (US) stimuli, a neural representation of the CS must be maintained over time to be associated with the later US. Usually, temporal associations are studied by investigating individual brain regions. It remains unknown, however, the effect of the interval at the network level, uncovering functional connections cooperating for the CS transient memory and its fear association. We investigated the functional network supporting temporal associations using a task in which a 5-s interval separates the contextual CS from the US (CFC-5s). We quantified c-Fos expression in forty-nine brain regions of male rats following the CFC-5s training, used c-Fos correlations to generate functional networks, and analyzed them by graph theory. Control groups were trained in contextual fear conditioning, in which CS and US overlap. The CFC-5s training additionally activated subdivisions of the basolateral, lateral, and medial amygdala; prelimbic, infralimbic, perirhinal, postrhinal, and intermediate entorhinal cortices; ventral CA1 and subiculum. The CFC-5s network had increased amygdala centrality and higher amygdala internal and external connectivity with the retrosplenial cortex, thalamus, and hippocampus. Amygdala and thalamic nuclei were network hubs. Functional connectivity among these brain regions could support CS transient memories and their association.

## Introduction

Some mnemonic processes, such as working memory and trace conditioning, rely on maintaining a transient neural representation of a stimulus without sensory input in its absence from the environment. In spatial working memory (SWM), a representation of a spatial cue is maintained during a delay to execute a goal-direct action^1,2^. In trace conditioning, a form of fear conditioning, a representation of a preceding conditioned stimulus (CS) is maintained over a trace interval to be associated with a posterior unconditioned stimulus (US)^3,4^. This transient memory enables stimuli, or stimulus and responses, to be associated when separated in time.

Memory conceptualizations propose that fear conditioning is supported by strengthening synaptic connections between neurons from different brain regions activated by the CS and US, forming a distributed brain network^5–7^. This concept is based on associations of stimuli overlapped in time, such as contextual fear conditioning (CFC), in which a contextual CS is associated with the US overlapped in time^7^. In associations of stimuli separated in time, the neuronal activity related to the CS's maintenance during the interval can be part of the functional network that encodes the memory, together with the neuronal activity related to associating this CS representation with the US, constituting a network of co-activated brain regions distinct from those supporting overlapped associations. Due to the time interval, temporal associations can engage different brain regions at the individual level of analysis and differential functional connections among them at the network level of analysis.

For instance, the lateral amygdala (LA)^8^, lateral entorhinal cortex (LEC)^9^, and perirhinal cortex (PER)^10^ have endogenous persistent-firing neurons, which continue to discharge in a self-sustained manner, a feature suitable for maintaining a transient neural representation of stimuli. Pretraining inactivation of the LA^11^, LEC^12^, or PER^13^ impaired trace conditioning. The prelimbic cortex (PL) also has persistent firing during time intervals^14^, which was required to encode trace conditioning^15^. It remains unknown, however, whether these brain regions engaged individually would also functionally interact, forming a neural network supporting temporal associations.

The basolateral amygdala (BLA) receives convergent information about the CS and US for their fear association^16^. The BLA has CS- and US-responsive neurons^17,18^, which have convergent activation^19,20^ and are required to encode associations separated and overlapped in time^4,16^. In temporal associations, brain regions supporting the CS's maintenance during the interval could be functionally connected with brain regions that associate it with the US, such as the BLA. Therefore, associations separated in time can recruit differential amygdala connectivity than associations overlapped in time.

We investigated the functional network underlying temporal associations. For this, we used the CFC-5s task in which a 5-s interval separates the context from the aversive US^21,22^. Trace conditioning can promote concomitantly associations separated (tone-US) and overlap (context-US) in time, given that only the tone is separated from the US. In CFC-5s, the context is the only CS and is separated from the US. A control group for associations that overlapped in time was trained in standard CFC. We observed the activity and interactivity of forty-nine brain regions related to temporal associations by quantifying the c-Fos expression following the CFC-5s training and built functional networks using brain regions' co-activation. Applying graph analysis, we characterized and compared functional networks underlying CFC-5s and CFC associations separated and overlapped in time.

Results showed that a 5-s interval between the context and the US altered the functional network of fear conditioning, increasing amygdala connectivity and network centrality. CFC-5s learning also activated eleven brain regions more than CFC learning. Present results characterize, for the first time, a functional network of temporal associations, which is relevant to understanding processes depending on transient memories and their associations, such as working memory.

## Results

### CFC-5s training activated eleven additional brain regions compared to CFC

**Figure 1:**
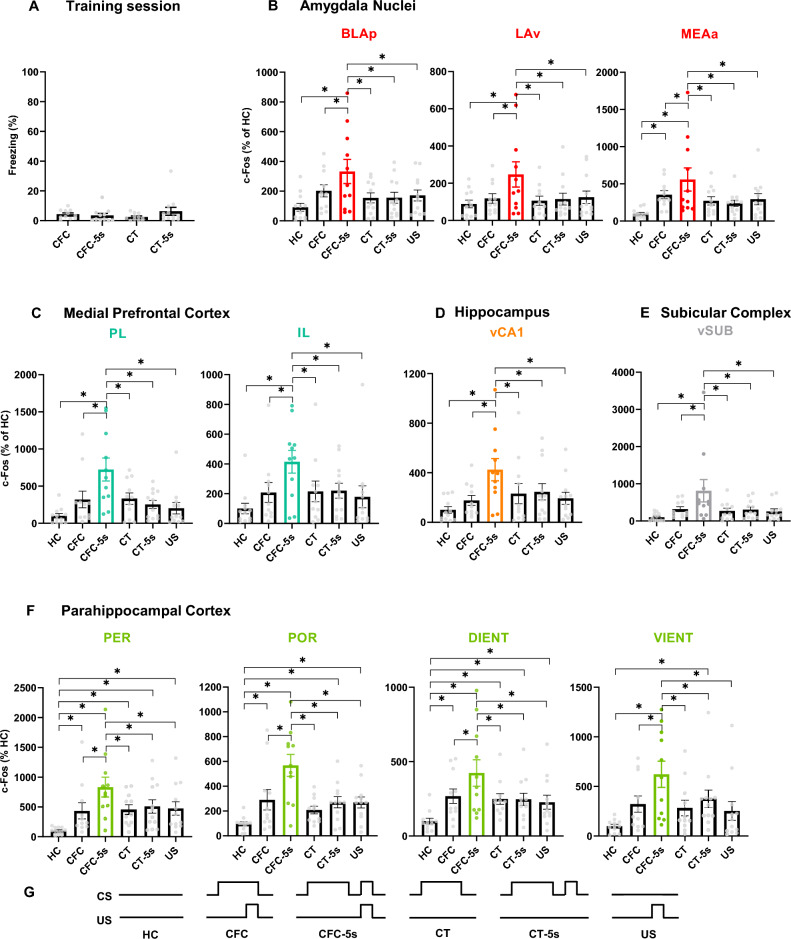
Learning-specific activity induced by CFC-5s training. (**A**) Freezing time among the CFC, CFC-5s, CT, and CT-5s groups in the training session. CFC-5s induced higher c-Fos expression than all the other experimental conditions in eleven specific regions, namely, (**B**) the basolateral posterior (**BLAp**), the lateral ventral (**LAv**), and the medial anterior (**MEAa**) amygdala nuclei; (**C**) the prelimbic (**PL**) and infralimbic (**IL**) cortices of the medial prefrontal cortex; (**D**) the ventral CA1 (**vCA1**) of the hippocampus; (**E**) the ventral subiculum (**vSUB**) of the subicular complex and (**F**) the perirhinal (**PER**), postrhinal (**POR**), dorsal intermediate entorhinal (**DIENT**) and ventral intermediate entorhinal (**VIENT**) cortices of the parahippocampal cortex. Table 1 shows the c-Fos expression in the other thirty-eight brain regions investigated. (**G**) Experimental design of the HC (n = 12), CFC (n = 11), CFC-5s (n = 11), CT (n = 11), CT-5s (n = 12), and US (n = 11) groups. **CFC:** contextual fear conditioning**; CFC-**5s**:** contextual fear conditioning with 5-s interval; **CS:** conditioned stimulus; **CT:** context; **HC**: homecage. **US**: unconditioned stimulus.

No group differed a priori regarding freezing responses (Fig. 1A). GZLM showed that the CFC-5s, CFC, CT, and CT-5s groups had similar freezing times in the training session (Wald = 2.384; degree of freedom = 3; *p* = 0.497). CFC-5s learning specifically engaged eleven brain regions (Fig. 1B–F, Table 1). The CFC-5s training induced activation of subdivisions of the medial prefrontal cortex (mPFC; the IL and PL); the amygdala nuclei (the BLAp, LAv, and MEAa), the hippocampal formation (the vCA1 and vSUB), the parahippocampal cortex (PHC; the PER and POR), and the lateral entorhinal cortex (LEC; the DIENT and VIENT). c-Fos expression was significantly higher in these eleven brain regions following the CFC-5s training than in all the other experimental conditions. These observations are supported by GZLM showing a significant group effect in the BLAp (W = 17.176; df = 5; *p* = 0.004), LAv (W = 12.899; df = 5; *p* = 0.024), MEAa (W = 20.596; df = 5; *p* = 0.001), PL (W = 30.214; df = 5; *p* = 0.001), IL (W = 14.610; df = 5; *p* = 0.012), vCA1 (W = 16.648; df = 5; *p* = 0.005), vSUB (W = 18.345; df = 5; *p* = 0.003), PER (W = 23.425; df = 5; *p* = 0.001), POR (W = 41.790; df = 5; *p* = 0.001), DIENT (W = 22.928; df = 5; *p* = 0.001), and VIENT (W = 20.756; df = 5; *p* = 0.001). LSD tests showed that the CFC-5s group had higher c-Fos expression than the CFC (BLAp *p* = 0.037; β = 0.792; LAv *p* = 0.011; β = 0.998; MEAa *p* = 0.050 β = 0.713; PL *p* = 0.001; β = 1.145; IL *p* = 0.018; β = 0.918; vCA1 *p* = 0.004; β = 1.109; vSUB *p* = 0.006; β = 1.034; PER *p* = 0.010; β = 0.915; POR *p* = 0.001; β = 1.202; DIENT *p* = 0.025; β = 0.823; VIENT *p* = 0.014; β = 0.920), CT (BLAp *p* = 0.005; β = 1.082; LAv *p* = 0.005; β = 1.089; MEAa *p* = 0.007; β = 0.998; PL *p* = 0.002; β = 1.112; IL *p* = 0.022; β = 0.883; vCA1 *p* = 0.024; β = 0.862; vSUB *p* = 0.003; β = 1.133; PER *p* = 0.016; β = 0.737; POR *p* = 0.001; β = 1.559; DIENT *p* = 0.012; β = 0.924; VIENT *p* = 0.005; β = 1.042), CT-5s (BLAp *p* = 0.004; β = 1.075; LAv *p* = 0.008; β = 1.023; MEAa *p* = 0.002; β = 1.114; PL *p* = 0.001; β = 1.334; IL *p* = 0.023; β = 0.861; vCA1 *p* = 0.033; β = 0.797; vSUB *p* = 0.004; β = 1.057; PER *p* = 0.033; β = 1.057; POR *p* = 0.001; β = 1.288; DIENT *p* = 0.010; β = 0.933; VIENT *p* = 0.037; β = 0.760), US (BLAp *p* = 0.008; β = 0.983; LAv *p* = 0.014; β = 0.946; MEAa *p* = 0.011; β = 0.924; PL *p* = 0.001; β = 1.480; IL *p* = 0.006; β = 1.044; vCA1 *p* = 0.006; β = 1.031; vSUB *p* = 0.002; β = 1.162; PER *p* = 0.019; β = 1.162; POR *p* = 0.001. β = 1.294; DIENT *p* = 0.004; β = 1.037; VIENT *p* = 0.002; β = 1.133), and HC (BLAp *p* = 0.001; β = 1.467; LAv *p* = 0.001; β = 1.223; MEAa *p* = 0.001; β = 1.595; PL *p* = 0.001; β = 1.771; IL *p* = 0.001; β = 1.394; vCA1 *p* = 0.001; β = 1.450; vSUB *p* = 0.001; β = 1.490; PER *p* = 0.001; β = 1.490; POR *p* = 0.001; β = 2.041; DIENT *p* = 0.001; β = 1.709; VIENT *p* = 0.001; β = 1.599) groups in these eleven brain regions. Although significantly lower than the CFC-5s expression, all groups had higher c-Fos expression than the HC in the DIENT (CFC *p* = 0.014; β = 0.886; CT *p* = 0.029; β = 0.786; CT-5s *p* = 0.027; β = 0.776; US *p* = 0.050; β = 0.672) and PER (CFC *p* = 0.028; β = 0.787; CT *p* = 0.019; β = 0.839; CT-5s *p* = 0.006; β = 0.962; US *p* = 0.012; β = 0.883). The CFC (*p* = 0.011; β = 0.839), CT-5s (*p* = 0.019; β = 0.752), and US (*p* = 0.020; β = 0.746) groups also had higher c-Fos expression than the HC in the POR; the CFC than the HC in the MEAa (*p* = 0.016; β = 0.882), and the CT-5s than the HC in the VIENT (*p* = 0.019; β = 0.838). Given that the CFC and CT-5s groups had higher activation of the DIENT, PER, and POR, although significative lower than the CFC-5s group, it could be interpreted that temporal learning, contextual fear conditioning, and the time interval activated these brain regions, which temporal learning activating more. Alternatively, contextual fear conditioning and the time interval could activate the brain regions, and they both combined activated more of them in the CFC-5s group in a cumulative effect.

**Table 1:**
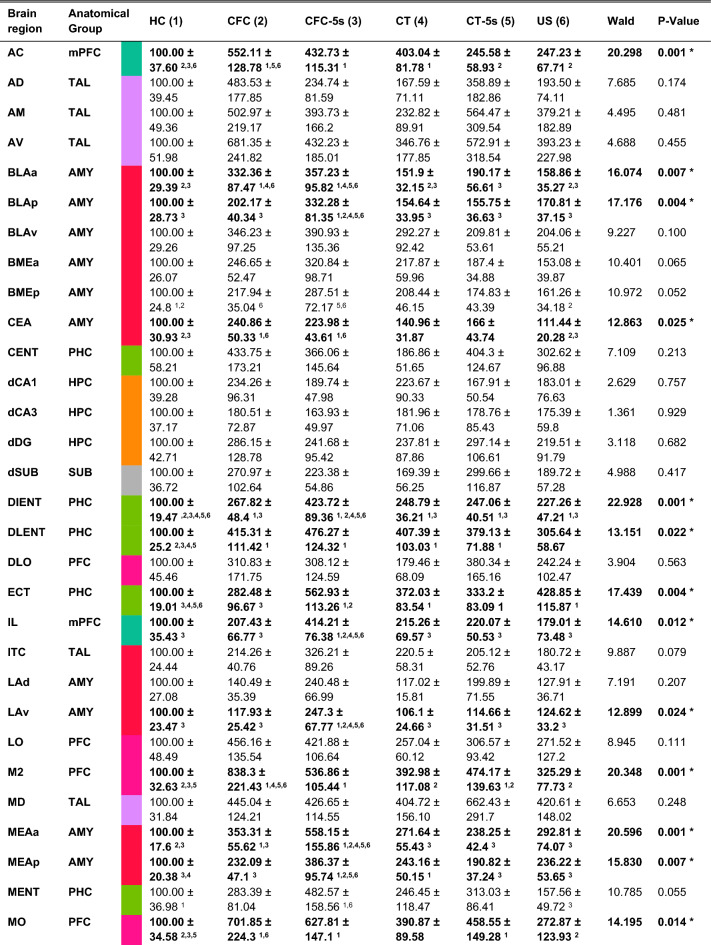

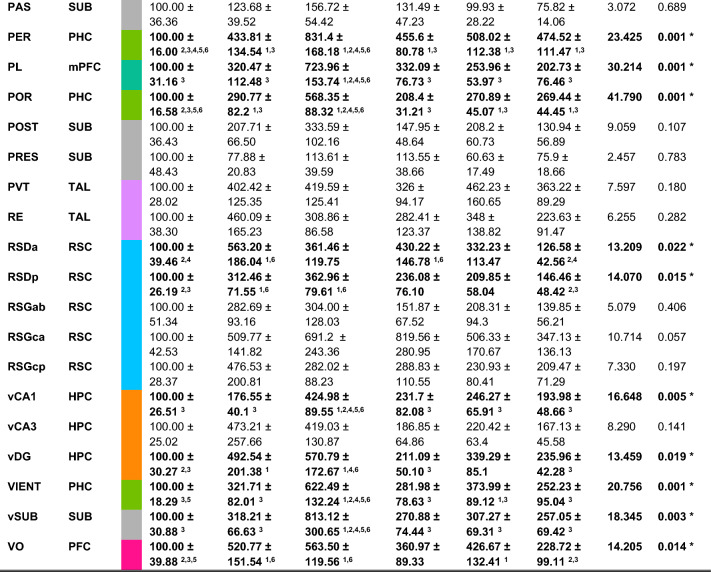
Quantification of c-Fos-positive cells following the CFC-5s training.

Mean ± standard error of c-Fos expression (% of the HC group) in the CFC (n = 11), CFC-5s (n = 11), CT (n = 11), CT-5s (n = 12), US (n = 12) and HC (n = 12) groups. Generalized Linear Model followed by LSD test. ^**1**^*p* < 0.050 compared to HC group; ^**2**^to CFC group; ^**3**^to CFC-5s group; ^**4**^to CT group; ^**5**^to CT-5s group and ^**6**^ to US group. Cingulate cortex (**AC**); anterodorsal thalamic nucleus (**AD**); anteromedial thalamic nucleus (**AM**); anteroventral thalamic nucleus (**AV**); basolateral amygdaloid nucleus, anterior (**BLAa**); basolateral amygdaloid nucleus, posterior (**BLAp**); basolateral amygdaloid nucleus, ventral (**BLAv**); basomedial amygdaloid nucleus, anterior (**BMEa**); basomedial amygdaloid nucleus, posterior (**BMEp**); caudomedial entorhinal cortex (**CENT**); central amygdaloid nucleus (**CEA**); dorsal CA1 region of hippocampus (**dCA1**); dorsal CA3 region of hippocampus (**dCA3**); dorsal dentate gyrus (**dDG**); dorsal intermediate entorhinal cortex (**DIENT**); dorsal lateral entorhinal cortex (**DLENT**); dorsal subiculum (**dSUB**); dorsolateral orbital cortex (**DLO**); ectorhinal cortex (**ECT**); infralimbic cortex (**IL**); intercalated amygdaloid nucleus (**ITC**); lateral amygdaloid nucleus, dorsal (**LAd**); lateral amygdaloid nucleus, ventral (**LAv**); lateral orbital cortex (**LO**); secondary motor cortex (**M2**); medial amygdaloid nucleus, anterior (**MEAa**); medial amygdaloid nucleus, posterior (**MEAp**); medial entorhinal cortex (**MENT**); medial orbital cortex (**MO**); mediodorsal thalamic nucleus (**MD**); parasubiculum (**PAS**); paraventricular thalamic nucleus (**PVT**); perirhinal cortex (**PER**); postrhinal cortex (**POR**); postsubiculum (**POST**); prelimbic cortex (**PL**); presubiculum (**PRES**); retrosplenial dysgranular cortex, anterior A30 (**RSDa**); retrosplenial dysgranular cortex, posterior (**RSDp**); retrosplenial granular cortex, A29ab (**RSGab**); retrosplenial granular cortex, anterior A29c (**RSGa**); retrosplenial granular cortex, posterior A29c (**RSGp**); reuniens thalamic nucleus (**RE**); ventral CA1 region of the hippocampus (**vCA1**); ventral CA3 region of the hippocampus (**vCA3**); ventral dentate gyrus (**vDG**); ventral intermediate entorhinal cortex (**VIENT**); ventral orbital cortex (**VO**); ventral subiculum (**vSUB**). Brain regions were categorized into nine anatomical groups to reflect the major brain subdivision of the prefrontal cortex (**PFC**); medial prefrontal cortex (**mPFC**); retrosplenial cortex (**RSC**); thalamus (**TAL**); amygdala (**AMY**); dorsal hippocampus (**DH**); ventral hippocampus (**VH**); subicular complex (**SUB**) and para-hippocampal cortex (**PHC**). See group names in Fig. 1.

In addition, the CFC-5s group had higher activation of the MEAp than all other groups except the CT group. GZLM showed a significant group effect in the MEAp (W = 15.830; df = 5; *p* = 0.007) and the LSD tests that the CFC-5s group had higher c-Fos expression than the CFC (*p* = 0.040; β = 0.786), CT-5s (*p* = 0.008; β = 0.997), US (*p* = 0.041; β = 0.765), and HC (*p* = 0.001; β = 1.460) groups.

All brain regions engaged in CFC learning were also involved in CFC-5s learning. Fear conditioning activated the AC, BLAa, CEA, M2, vDG, and RSDp. In these brain regions, c-Fos expression was similar in CFC and CFC-5s groups and higher than in other control groups. These observations are supported by GZLM showing a significant group effect in the AC (W = 20.298; df = 5; *p* = 0.001), BLAa (W = 16.074; df = 5; *p* = 0.007), CEA (W = 12.863; df = 5; *p* = 0.025), M2 (W = 20.348; df = 5; *p* = 0.001), vDG (W = 13.459; df = 5; *p* = 0.019), and RSDp (W = 14.070; df = 5; *p* = 0.015). LSD tests showed that the CFC and CFC-5s groups had higher c-Fos expression than the HC group in the AC (CFC *p* = 0.001; β = 1.451; CFC-5s *p* = 0.003; β = 1.072), BLAa (CFC *p* = 0.005; β = 1.062; CFC-5s *p* = 0.002; β = 1.176), CEA (CFC *p* = 0.006; β = 1.055; CFC-5s *p* = 0.015; β = 0.929), M2 (CFC *p* = 0.001; β = 1.574; CFC-5s *p* = 0.011; β = 0.931), vDG (CFC *p* = 0.011; β = 0.974; CFC-5s *p* = 0.002; β = 1.169), and RSDp (CFC *p* = 0.011; β = 0.969; CFC-5s *p* = 0.002; β = 1.199). The CFC and CFC-5s groups also had higher c-Fos expression than the US group in the BLAa (CFC *p* = 0.034; β = 0.793; CFC-5s *p* = 0.016; β = 0.907), CEA (CFC *p* = 0.011; β = 0.970; CFC-5s *p* = 0.028; β = 0.843), and RSDp (CFC *p* = 0.046; β = 0.757; CFC-5s *p* = 0.009; β = 0.988), and than the CT group in the BLAa (CFC *p* = 0.031; β = 0.825; CFC-5s *p* = 0.014; β = 0.939). In addition, the CFC group had higher c-Fos expression in the M2 than the CT (*p* = 0.011; β = 0.949), CT-5s (*p* = 0.034; β = 0.776), and US (*p* = 0.003; β = 1.094) groups, and in the AC than the CT-5s (*p* = 0.008; β = 0.973) and US (*p* = 0.008; β =) groups. The CFC-5s group also had higher c-Fos expression than the US in the vDG (*p* = 0.029; β = 0.831), the CT in the vDG (*p* = 0.022; β = 0.893), and the CT-5s in the BLAa (*p* = 0.042; β = 0.764). Finally, the CT group had higher c-Fos expression than the HC in the AC (*p* = 0.007; β = 0.978), and the CT-5s than the HC in the M2 (*p* = 0.026; β = 0.798).

Regardless of its fear association, contextual learning activated the DLENT, MO, and VO. In these brain regions, groups conditioned and pre-exposed to context had a similar c-Fos expression and higher than in the US or HC groups. GZLM showed a significant group effect in the DLENT (W = 13.151; df = 5; *p* = 0.022), MO (W = 14.195; df = 5; *p* = 0.014), and VO (W = 14.205; df = 5; *p* = 0.014). LSD tests showed that the CFC (*p* = 0.007; β = 1.023), CFC-5s (*p* = 0.001; β = 1.221) CT (*p* = 0.009; β = 0.998), and CT-5s (*p* = 0.015; β = 0.906) groups had higher c-Fos expression than the HC in the DLENT; the CFC, CFC-5s, and CT-5s groups than the HC in the MO (CFC *p* = 0.001; β = 1.217; CFC-5s *p* = 0.005; β = 1.067; CT-5s *p* = 0.050; β = 0.725) and VO (CFC *p* = 0.005; β = 1.070; CFC-5s *p* = 0.002; β = 1.178; CT-5s *p* = 0.025; β = 0.830); the CFC (*p* = 0.050; β = 0.742) and CFC-5s (*p* = 0.025; β = 0.851) groups also than the US in the VO, and the CFC than the US in the MO (*p* = 0.022; β = 0.867).

Contextual exposure continuously in time seems to engage the RSDa. C-Fos expression in the RSDa was higher in the CFC and CT groups than in the US and HC groups. GZLM showed a significant group effect in the RSDa (W = 13.209; df = 5; *p* = 0.022), and the LSD tests that the CFC and CT groups had higher c-Fos expression than the HC (CFC *p* = 0.003; β = 1.146; CT *p* = 0.031; β = 0.825) and US (CFC *p* = 0.006; β = 1.056; CT *p* = 0.050; β = 0.735) groups in the RSDa.

The ECT was engaged indiscriminately in different experimental conditions. GZLM showed a group effect in the ECT (W = 17.439; df = 5; *p* = 0.004) and the LSD tests that the CFC-5s (*p* = 0.001; β = 1.465), CT (*p* = 0.017; β = 0.885), CT-5s (*p* = 0.035; β = 0.767), and US (0.004; β = 1.058) groups had higher c-Fos expression than the HC group, and the CFC-5s also than the CFC (*p* = 0.025; β = 0.852). The other twenty-six brain regions investigated had no significant changes among the groups. The statistical analyses of the 49 brain regions are fully described in Table 1. Supplementary Fig. S1 shows representative images of c-Fos expression. Overall, CFC-5s was mediated by activity in distinct brain regions, partially matching the CFC.

### CFC-5s strengthened amygdala connectivity internally and externally with the retrosplenial cortex, thalamus, and hippocampus

**Figure 2:**
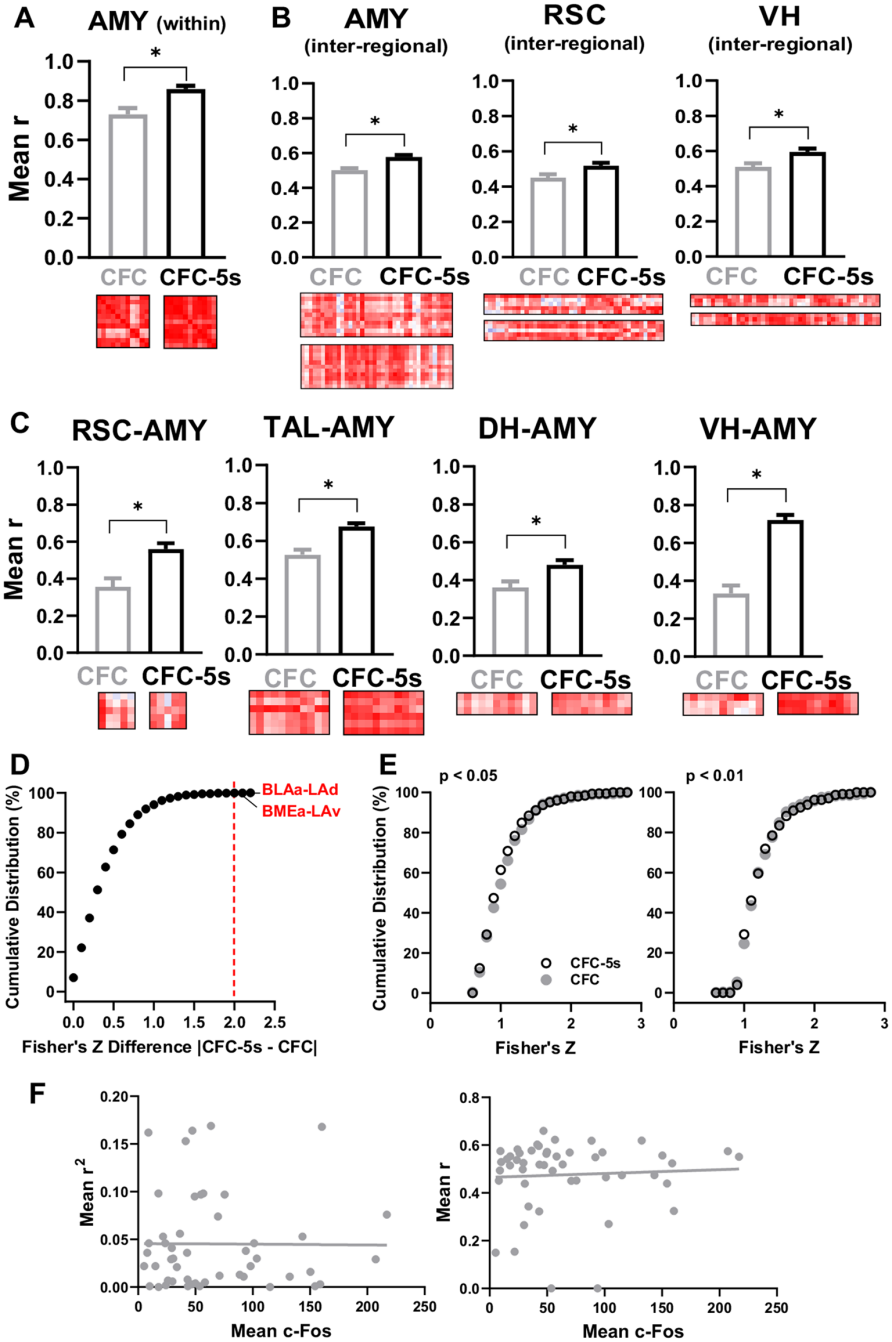
Major brain subdivisions with higher internal and external functional connectivity following the CFC-5s training. (**A**) Comparison of connectivity within each anatomical group. CFC-5s had a higher mean correlation coefficient within the amygdala nuclei (**AMY**). (**B**) Comparison of inter-regional connectivity. The CFC-5s had a higher mean correlation coefficient between the AMY, the retrosplenial cortices (**RSC**), the ventral hippocampus (**VH**), and the other corresponding anatomical groups. (**C**) Comparison of connectivity between pairs of anatomical groups. The CFC-5s had a higher mean correlation coefficient between the RSC and AMY, the thalamic nuclei (**TAL**) and AMY, the dorsal hippocampus (**DH**) and AMY, and the VH and AMY. Generalized linear models, * indicates *p* < 0.050. Mean (± standard error) of correlation coefficients. (**D**) Cumulative distribution of the Fisher's Z Differences (correlation coefficients normalized by the Z Fisher transformation) between the CFC and CFC-5s groups. The red dashed lines indicate z-scores ≥ 2, values considered significant at α = 0.050. (**E**) Cumulative distributions of the Fisher's Z in the CFC (grey circles) and CFC-5s (black circles) functional networks using *p* < 0.05 or *p* < 0.01 threshold levels. Two-sample Kolmogorov–Smirnov test. See all results in Supplementary Table S1. (**F**) Correlations between the mean c-Fos expression and the mean correlation coefficient or mean r^2^ (from Linear Regressions of mean c-Fos expression predicting mean correlation coefficient in each brain region) of all brain regions. **CFC:** contextual fear conditioning**; CFC-**5s**:** contextual fear conditioning with 5-s interval; **BLAa**: basolateral amygdaloid nucleus, anterior; **BMEa**: basomedial amygdaloid nucleus, anterior; **LAd**: lateral amygdaloid nucleus, dorsal; **LAv**: lateral amygdaloid nucleus, ventral. See group names in Fig. 1.

**Figure 3:**
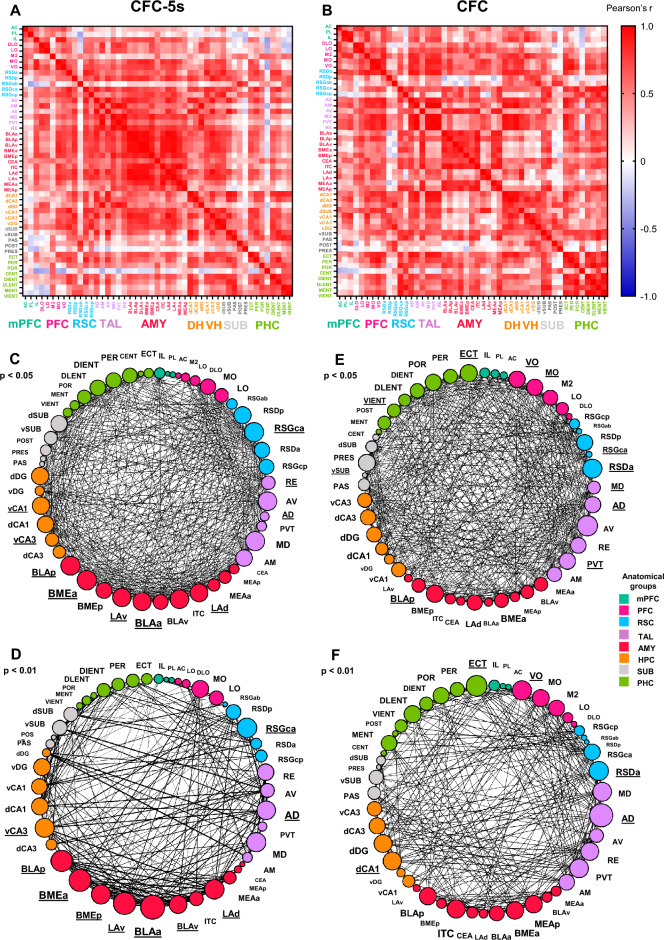
Correlation matrices and functional networks of CFC and CFC-5s groups. The correlation coefficients of c-Fos expression between each pair of brain regions were computed in the CFC-5s (**A**) and the CFC (**B**) groups. Colors reflect correlation strength (scale, right). Functional networks were generated by selecting the significant positive correlation coefficients. Two threshold levels were applied (*p* < 0.05 and *p* < 0.01), generating two functional networks for the CFC-5s (**C**,**D**) and the CFC (**E**,**F**) groups. Nodes represent brain regions with a size proportional to their number of functional connections (degree). Colors reflect the anatomical group the brain region belongs (scale, right). Brain regions posteriorly identified as hubs were subscripted. Edges between nodes represented correlation coefficients above the threshold level and were thought to reflect functional connections. The thickness of the edges was proportional to the strength of the correlation. See group names in Fig. 1. See an overview and comparison of CFC and CFC-5s functional networks in Supplementary Table S2.

We computed the correlation coefficients (Pearson's r) of the mean c-Fos expression between all pairs of brain regions for the CFC and CFC-5s groups, generating a complete set of correlation coefficients (Fig. 3A,B). We next categorized the forty-nine brain regions into nine anatomical groups to reflect major brain subdivisions. We calculated the average of the correlation coefficients (connectivity) within each anatomical group (internal connectivity), between one anatomical group and the remained ones (external connectivity), or between all the pairs of anatomical groups (Fig. 2 and Supplementary Table S1). This approach considers that all correlation coefficients, despite their weight, could be functionally relevant. GZLM showed that the CFC-5s group had higher mean correlation coefficients than the CFC group within the amygdala nuclei (W = 9.475; df = 1; *p* = 0.002; β = 0.597; Fig. 2A) and between the amygdala nuclei (W = 20.332; df = 1; *p* = 0.001; β = 0.272), the retrosplenial cortex (RSC, W = 6.340; df = 1; *p* = 0.012; β = 0.226) or the ventral hippocampus (VH, W = 8.409; df = 1; *p* = 0.004; β = 0.333) and other brain regions (Fig. 2B). The CFC-5s group also had higher mean correlation coefficients than the CFC group between the amygdala nuclei and the RSC (W = 12.963; df = 1; *p* = 0.001; β = 0.645), the thalamic nuclei (W = 21.973; df = 1; *p* = 0.001; β = 0.753); the dorsal hippocampus (DH, W = 9.020; df = 1; *p* = 0.003; β = 0.866) or the VH (W = 60.869; df = 1; *p* = 0.001; β = 1.375). CFC-5s ' higher connectivity results are highlighted (Fig. 2A–C). All results are shown in Supplementary Table S2.

We next compared each correlation coefficient between the CFC and CFC-5s groups by computing their normalized difference (Fisher's Z difference). Results showed that the CFC-5s group had a significantly stronger correlation than the CFC group between the BMEa and the LAv (Fisher's Z difference = 2.000; CFC-5s r = 0.981; CFC r = 0.345) and between the BLAa and the LAd (Fisher's Z difference = 2.200; CFC-5s r = 0.981; CFC r = 0.324; Fig. 2D). Overall, CFC-5s strengthened internal and external amygdala connectivity

Linear Regressions showed that the c-Fos expression was not a significant factor in explaining the strength of the correlation coefficients in any brain region (AC r^2^ = 0.168; *p* = 0.058; AD r^2^ = 0.003; *p* = 0.819; AM r^2^ = 0.164 *p* = 0.061; AV r^2^ = 0.005; *p* = 0.756; BLAa r^2^ = 0.004; *p* = 0.309; BLAp r^2^ = 0.098; *p* = 0.157; BLAv r^2^ = 0.098; *p* = 0.157; BMEa r^2^ = 0.007; *p* = 0.707; BMEp r^2^ = 0.029; *p* = 0.451; CA1d r^2^ = 0.046; *p* = 0.337; CA1v r^2^ = 0.002; *p* = 0.841; CA3d r^2^ = 0.001; *p* = 0.887; CA3v r^2^ = 0.001; *p* = 0.967; CEA r^2^ = 0.095; *p* = 0.164; CENT r^2^ = 0.053; *p* = 0.302; dDG r^2^ = 0.162; *p* = 0.064; DIENT r^2^ = 0.016; *p* = 0.576; DLENT r^2^ = 0.003; *p* = 0.794; DLO r^2^ = 0.021; *p* = 0.524; dSUB r^2^ = 0.022; *p* = 0.505; ECT r^2^ = 0.022; *p* = 0.508; IL r^2^ = 0.030; *p* = 0.440; ITC r^2^ = 0.056; *p* = 0.289; LAd r^2^ = 0.046; *p* = 0.338; LAv r^2^ = 0.041; *p* = 0.366; LO r^2^ = 0.046; *p* = 0.341; M2 r^2^ = 0.001; *p* = 0.937; MD r^2^ = 0.153; *p* = 0.072; MEAa r^2^ = 0.097; *p* = 0.159; MEAp r^2^ = 0.097; *p* = 0.159; MENT r^2^ = 0.012; *p* = 0.623; MO r^2^ = 0.076; *p* = 0.214; PAS r^2^ = 0.036; *p* = 0.398; PER r^2^ = 0.011; *p* = 0.638; PL r^2^ = 0.030; *p* = 0.440; POR r^2^ = 0.053; *p* = 0.302; POST r^2^ = 0.008; *p* = 0.701; PRES r^2^ = 0.022; *p* = 0.508; PVT r^2^ = 0.169; *p* = 0.058; RE r^2^ = 0.036; *p* = 0.400; RSDa r^2^ = 0.011; *p* = 0.642; RSDp r^2^ = 0.001; *p* = 0.919; RSGab r^2^ = 0.006; *p* = 0.731; RSGca r^2^ = 0.013; *p* = 0.613; RSGcp r^2^ = 0.001; *p* = 0.883; vDG r^2^ = 0.001; *p* = 0.386; VIENT r^2^ = 0.038; *p* = 0.386; VO r^2^ = 0.029; *p* = 0.937; vSUB r^2^ = 0.074; *p* = 0.219). There was not a significant correlation between the r^2^ and the c-Fos expression (r = -0.008; *p* = 0.959) nor between the correlation coefficients and the c-Fos expression (r = 0.057; *p* = 0.696), suggesting that the magnitude of the c-Fos expression was not contributing to differences in the strength of the correlation coefficients (Fig. 2F).

### Generation of CFC and CFC-5s functional networks

We selected the significant positive correlation coefficients to build functional networks for the CFC-5s (Fig. 3C,D) and CFC (Fig. 3E,F) groups. We constructed two functional networks for each group, using two thresholds of p-values (*p* < 0.050 and *p* < 0.010) to evaluate whether and which network properties were stable, independent of the p-value used. Although our criteria did not include negative correlations, there were no significantly negative ones. Functional networks consisted of nodes (brain regions) connected by undirected edges (correlation coefficients above the p-value threshold level).

The CFC-5s and CFC functional networks had 49 brain regions connected by 930 and 946 edges (*p* < 0.050) or 46 and 49 brain regions connected by 484 and 562 edges (*p* < 0.010), respectively. The graph density (how many edges the network had, from the total of possible ones) was 0.790 (*p* < 0.050) and 0.469 (*p* < 0.010) for the CFC-5s, and 946 (*p* < 0.050) and 0.479 (*p* < 0.010) for the CFC functional network. A complete network would have a density of 1. Brain regions were connected up to 5 edges in the CFC-5s and up to 4 (*p* < 0.010) and 5 (*p* < 0.050) edges in the CFC functional networks. The average distance (average path length) was 1.755 and 1.678 edges (*p* < 0.050) and 2.126 and 2.222 edges (*p* < 0.010) in the CFC-5s and CFC functional networks, respectively (Supplementary Table S2).

### CFC and CFC-5s functional networks had similar organization and topological properties

The CFC and CFC-5s networks had similar average degrees, weighted degrees, cluster coefficients, and global and nodal efficiencies, as shown by between-group comparisons^23,24^. These measures significantly decreased in both groups in the functional networks using the more conservative threshold (*p* < 0.010) in within-group comparisons to the less conservative threshold (*p* < 0.050, Supplementary Table S2).

Between groups comparisons showed that the CFC and CFC-5s networks using the less (*p* < 0.050) or more (*p* < 0.010) conservative thresholds had a similar average degree (GZLM *p* < 0.050 networks: W = 0.043; df = 1; *p* = 0.836; GZLM *p* < 0.010 networks: W = 0.649; df = 1; *p* = 0.421); average weighted degree (GZLM *p* < 0.050 networks: W = 0.258; df = 1; *p* = 0.612; GZLM *p* < 0.010 networks: W = 0.653; df = 1; *p* = 0.419); average cluster coefficient (GZLM *p* < 0.050 networks: W = 0.318; df = 1; *p* = 0.573; GZLM *p* < 0.010 networks: W = 2.334; df = 1; *p* = 0.127); global efficiency (GZLM *p* < 0.050 networks: W = 0.190; df = 1; *p* = 0.663; GZLM *p* < 0.010 networks: W = 2.834; df = 1; *p* = 0.092), and nodal efficiency (GZLM *p* < 0.050 networks: W = 0.235; df = 1; *p* = 0.628; GZLM *p* < 0.010 networks: W = 0.803; df = 1; *p* = 0.370). The distribution of global efficiencies was distinct in the CFC and CFC-5s networks (K-S *p* < 0.050 networks: *p* = 0.037; K-S *p* < 0.010 networks: *p* = 0.001).

Within-group comparisons showed that the functional networks using the less conservative thresholds (*p* < 0.050) had a higher average degree (GZLM CFC networks: W = 23.533; df = 1; *p* = 0.001; GZLM CFC-5s networks: W = 35.542; df = 1; *p* = 0.001); average weighted degree (GZLM CFC networks: W = 14.496; df = 1; *p* = 0.001; GZLM CFC-5s networks: W = 26.620; df = 1; *p* = 0.001); average clustering coefficient (GZLM CFC networks: W = 4.854; df = 1; *p* = 0.028; GZLM CFC-5s networks: W = 4.583; df = 1; *p* = 0.032); global efficiency (GZLM CFC networks: W = 47.208; df = 1; *p* = 0.001; GZLM CFC-5s networks: W = 165.556; df = 1; *p* = 0.001), and nodal efficiency (GZLM CFC networks: W = 17.308; df = 1; *p* = 0.001; GZLM CFC-5s networks: W = 45.439; df = 1; *p* = 0.001) than the networks using the more conservative thresholds (*p* < 0.010), in both CFC and CFC-5s groups, which may reflect the decrease of edges (functional connections). In addition, the distribution of the average degree (K-S CFC: *p* = 0.001; K-S CFC-5s: *p* = 0.001), average weighted degree (K-S CFC: p—0.001; K-S CFC-5s: *p* = 0.001), clustering coefficients (K-S CFC: *p* = 0.001; K-S CFC-5s: *p* = 0.001), global efficiency (K-S CFC: *p* = 0.001; K-S CFC-5s: *p* = 0.001) and nodal efficiency (K-S CFC: *p* = 0.001; K-S CFC-5s: *p* = 0.001) were distinct in the networks using more (*p* < 0.050) and less (*p* < 0.010) conservative thresholds, in both CFC and CFC-5s groups. All results are shown in Supplementary Table S2.

Both functional networks had similar edge properties, with an equal distribution of the strength of the connections. The K-S test showed that there was no difference in the Z Fisher's distribution between the CFC and CFC-5s networks generated by the less (*p* = 0.399) or more (*p* = 0.381) conservative thresholds (Fig. 2E).

### Thalamic nuclei are connector hubs in the CFC-5s functional networks

**Figure 4:**
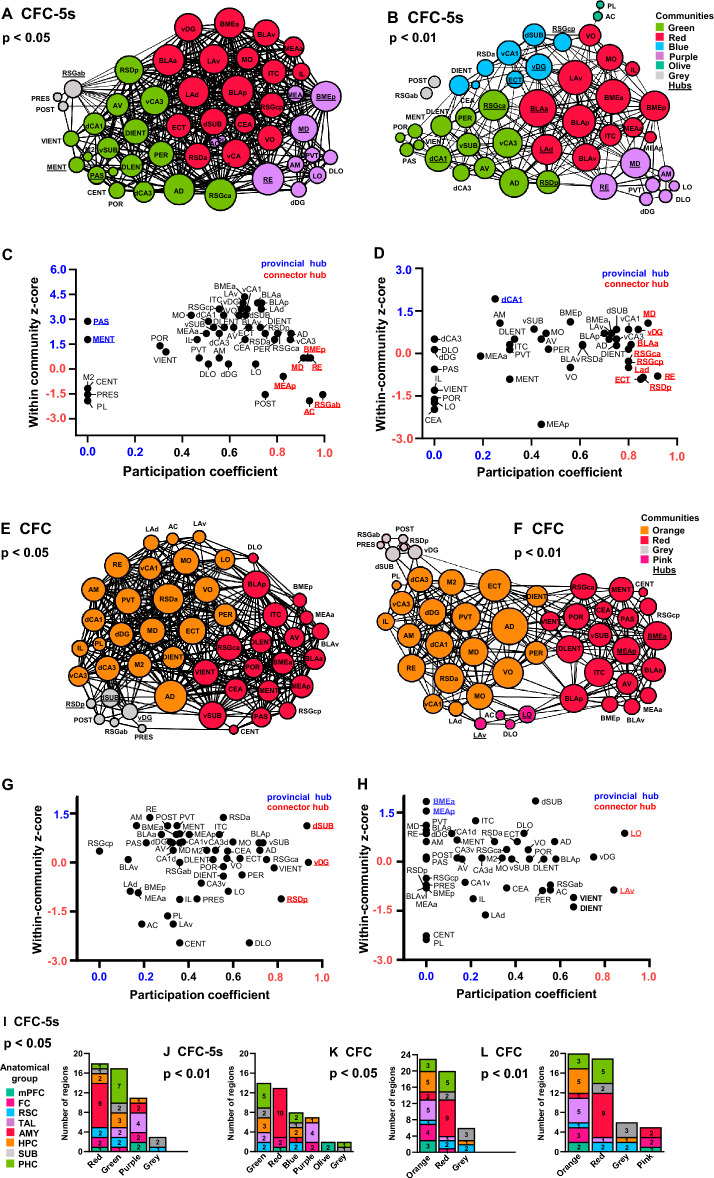
Community structure of the CFC-5s and CFC functional networks. CFC-5s and CFC networks (*p* < 0.05) in (**A**,**E**) and CFC-5s and CFC networks (*p* < 0.01) in (**B**,**F**) are subdivided into communities. Communities were represented in distinct colors (scale, right), indicating the anatomical group with the greatest participation. Brain regions identified as provincial or connector hubs were subscripted. WC (within-community) z-score and PC (participation coefficient) for each brain region of the CFC-5s and CFC networks using *p* < 0.05 (**C**,**G**) and *p* < 0.01 (**D**,**H**) thresholds. Provincial hubs (WC z-score ≥ 1.5 and PC ≤ 0.3) are in blue and connector hubs (PC ≥ 0.8 and WC z-scores ≤ 1.5) are in red. Number of brain regions from each anatomical group (represented by colors, scale on the left) in each community of the CFC-5s network using *p* < 0.05 (**I**) or *p* < 0.01 (**J**)**,** and in each community of the CFC network using *p* < 0.05 (**K**) or *p* < 0.01 (**L**)**.** We described the full name of the brain regions in Table 1. See group names in Fig. 1.

We decomposed the functional networks into communities using modularity optimization^25^. Communities consist of highly interconnected nodes with sparse functional connections with nodes from other communities and may represent functional modules^26^. The CFC-5s networks had communities with an over-representation of the amygdala nuclei, the thalamic nuclei, and the hippocampal formation (PHC, hippocampus, and subicular complex). Their proportions in these communities were qualitatively higher than those observed in the network as a whole (Fig. 4A–D). The CFC networks had one community with an over-representation of the amygdala nuclei and one of the hippocampus and the thalamic nuclei (Fig. 4E–H). The major differences were that the CFC-5s networks had one community over-representing the thalamus and another, the PHC. In contrast, the thalamic nuclei and hippocampus were in the same community, and the PHC spread more in CFC networks. Both the CFC and CFC-5s functional networks had a small community consisting of the SUB and the RSC.

We next calculated how well a brain region was connected in its community and with other communities using the within-community (WC) z-score and participation coefficient (PC) measures, respectively. WC z-score is a node's normalized number of connections inside its community. Positive values indicate higher WC connections than the community's mean. PC is calculated by subtracting from 1 the ratio between the number of WC connections and the total connections of a node. Values closer to 1 indicate more connections across the communities^26^. Brain regions with WC z-scores ≥ 1.5 and PC ≤ 0.3 were considered exclusively provincial hubs, essential for the interaction inside the community^27^. Brain regions with PC ≥ 0.8 and WC z-score ≤ 1.5 were considered solely connector hubs, essential to mediate community interactions^27^. The nuclei mediodorsal of the thalamus (MD; PC = 0.912; WC z-score = 0.670 in the *p* < 0.050; PC = 0.880; WC z-score = 1.069 in the *p* < 0.010 network) and reuniens of the thalamus (RE; PC = 0.941; WC z-score = 0.670 in the *p* < 0.050; PC = 0.920; WC z-score = -0.802 in the *p* < 0.010 network) were stable connector hubs in the CFC-5s functional networks, whereas there was no stable provincial hub (Fig. 4A,B). There were no stable connector or provincial hubs in the CFC functional networks (Fig. 4C,D). Although not stable across the networks, some amygdala nuclei were identified as connector hubs in the CFC-5s networks (BMEp PC = 0.929; WC z-score = 0.670 in the *p* < 0.050 network; MEAp PC = 0.826; WC z-score -0.435; BLAa PC = 0.810; WC z-score = 0.309; LAd PC = 0.800; WC z-score = -0.495 in the *p* < 0.010 network) and some as provincial hubs in the CFC network (BMEa PC = 0.000; WC z-score = 1.835; MEAp PC = 0.000; WC z-score = 1.542 in the *p* < 0.010 network), suggesting possible distinctive roles of the amygdala nuclei in CFC and CFC-5s networks (Fig. 4A–D).

### Amygdala nuclei are hubs in the CFC-5s functional networks

**Figure 5:**
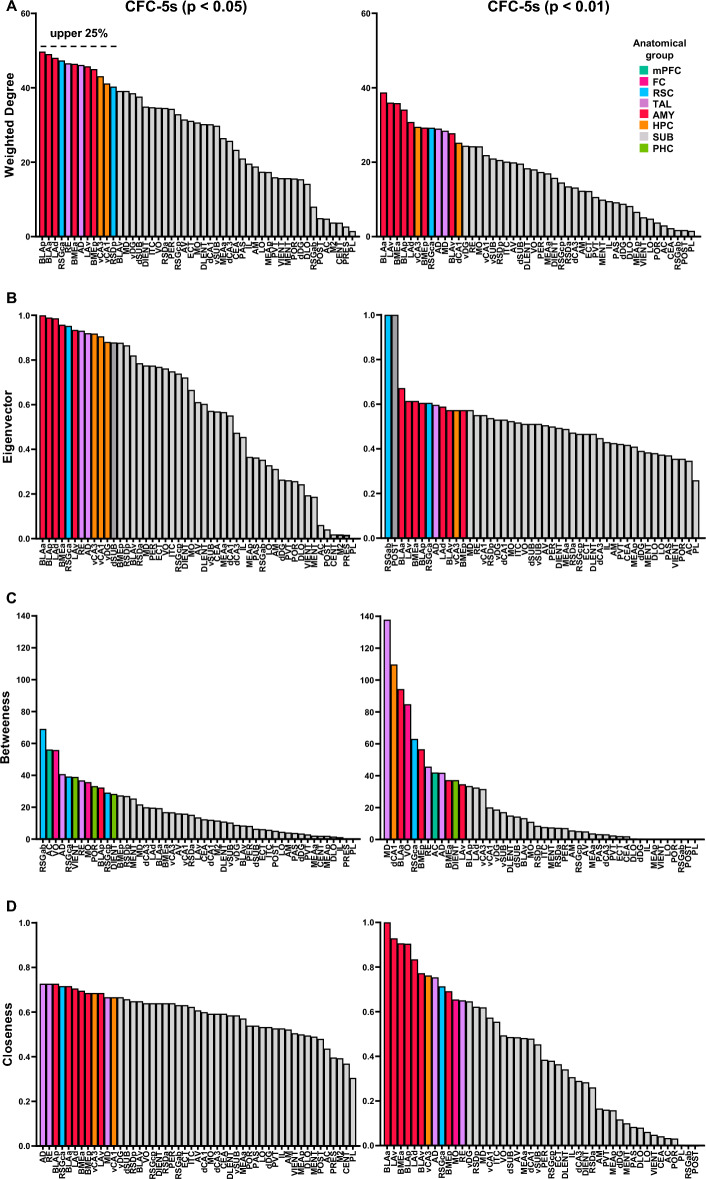
Brain regions ranked by their values in the centrality measures in the CFC-5s functional networks. Ranking for the weighted degree (**A**)**,** eigenvector (**B**)**,** betweenness (**C**)**,** and closeness (**D**) in the CFC-5s functional networks using *p* < 0.05 (left) or *p* < 0.01 (right) thresholds. Colored bars represent the upper 25% of the brain regions, considered high centrality nodes. Colors reflect the anatomical group the brain region belongs (scale, right). **CFC-**5s**:** contextual fear conditioning with a 5-s interval. See brain regions ranked by their values in the centrality measures in the CFC functional networks in Supplementary Figure S2.

Centrality measures assessed the importance of individual nodes. We calculated four measures of centrality, two based on the degree (number of edges), the weighted degree (Wdg), and the eigenvector (Evc), and two based on shortest paths, the betweenness (Bet) and closeness (Clo). We ranked the brain regions in each measure according to their values in decrescent order. Colored bars represent the upper 25% of the brain regions, considered high centrality nodes. High centrality nodes of CFC-5s functional networks are shown in Fig. 5 and of CFC functional networks in Supplementary Fig. S2.

The Wdg measures the number of edges of each node, weighted by their strength (correlation coefficient)^28^. The amygdala nuclei had higher Wdg in the CFC-5s and the thalamic nuclei in the CFC functional networks. Only AD was highly central in both groups. The RSGca (47.329 in the *p* < 0.050; 29.264 in the *p* < 0.010 network), AD (46.131 in the *p* < 0.050; 29.022 in the *p* < 0.010 network), BLAa (49.083 in the *p* < 0.050; 38.716 in the *p* < 0.010 network), BLAp (49.737 in the *p* < 0.050; 34.130 in the *p* < 0.010 network), BMEa (46.459 in the *p* < 0.050; 35.878 in the *p* < 0.010 networks), BMEp (45.050 in the *p* < 0.050; 29.306 in the *p* < 0.010 network), LAd (48.054 in the *p* < 0.050; 30.805 in the *p* < 0.010 network), LAv (45.784 in the *p* < 0.050 network; 36.034 in the *p* < 0.010 network), and vCA3 (43.105 in the *p* < 0.050; 29.502 in the *p* < 0.010 network) were stable nodes with a higher Wdg in the CFC-5s functional networks (Fig. 5A).

The RSDa (49.627 in the *p* < 0.050; 30.516 in the *p* < 0.010 network), the VO (40.780 in the *p* < 0.050; 30.498 in the *p* < 0.010 network), the AD (53.182 in the *p* < 0.050; 37.944 in the *p* < 0.010 network), the MD (40.648 in the *p* < 0.050; 29.844 in the *p* < 0.010 network), the PVT (41.380 in the *p* < 0.050; 29.690 in the *p* < 0.010 network), the RE (39.306 in the *p* < 0.050; 28.064 in the *p* < 0.010 network), and the ECT (43.864 in the *p* < 0.050; 33.492 in the *p* < 0.010 network) were stables nodes with a higher Wdg in the CFC functional networks (Supplementary Fig. S2).

The Evc measures a node's centrality based on its neighboring nodes' degree^29^. All previous brain regions, except the BMEp, were stable nodes with higher Evc in the CFC-5s functional networks (Fig. 5B; RSGca 0.952 in the *p* < 0.050; 0.606 in the *p* < 0.010 network; AD 0.920 in the *p* < 0.050; 0.597 in the *p* < 0.010 network; BLAa 1.000 in the *p* < 0.050; 0.672 in the *p* < 0.010 network; BLAp 0.989 in the *p* < 0.050; 0.606 in the *p* < 0.010 network; BMEa 0.958 in the *p* < 0.050; 0.614 in the *p* < 0.010 network; LAd 0.986 in the *p* < 0.050; 0.589 in the *p* < 0.010 network; LAv 0.935 in the *p* < 0.050; 0.614 in the *p* < 0.010 network; vCA3 0.918 in the *p* < 0.050; 0.573 in the *p* < 0.010 network).

All previous brain regions, except the RE and additionally the MO, were stable nodes in the CFC functional networks (Supplementary Fig. S2; RSDa 0.931 in the *p* < 0.050; 0.769 in the *p* < 0.010 network; VO 0.817 in the *p* < 0.050; 0.832 in the *p* < 0.010 network; AD 1.000 in the *p* < 0.050 and *p* < 0.010 networks; MD 0.782 in the *p* < 0.050; 0.778 in the *p* < 0.010 network; PVT 0.796 in the *p* < 0.050; 0.783 in the *p* < 0.010 network; MO 0.832 in the *p* < 0.050; 0.670 in the *p* < 0.010 network; ECT 0.869 in the *p* < 0.050; 0.893 in the *p* < 0.010 network).

The Bet measures the shortest path (edges) between two nodes that pass through a node^28^. The AC (56.239 in the *p* < 0.050; 42.000 in the *p* < 0.010 network), VO (55.913 in the *p* < 0.050; 84.798 in the *p* < 0.010 network), AD (40.767 in the *p* < 0.050; 41.832 in the *p* < 0.010 network), RSGca (39.330 in the *p* < 0.050; 63.097 in the *p* < 0.010 network), and DIENT (28.377 in the *p* < 0.050; 37.222 in the *p* < 0.010 network) were stable nodes with higher Bet in the CFC-5s networks (Fig. 5C).

The M2 (24.461 in the *p* < 0.050; in the *p* < 0.010 network) LO (34.436 in the *p* < 0.050; 51.713 in the *p* < 0.010 network) RSDa (54.995 in the *p* < 0.050; 85.520 in the *p* < 0.010 network), AD (45.525 in the *p* < 0.050; 117.424 in the *p* < 0.010 network), BLAp (48.126 in the *p* < 0.050; 82.696 in the *p* < 0.010 network), dCA3 (36.672 in the *p* < 0.050; 62.012 in the *p* < 0.010 network), vDG (25.366 in the *p* < 0.050; 96.076 in the *p* < 0.010 network), and dSUB (44.051 in the *p* < 0.050; 113.612 in the *p* < 0.010 network) were stables nodes with higher Bet in the CFC networks (Supplementary Fig. S2).

The Clo measures the average length of the shortest paths between a node and the others^28^. The brain regions with higher Wdg centralities also had the highest Clo centralities in the CFC-5s and CFC functional networks. The RSGca (0.716 in the *p* < 0.050; 0.713 in the *p* < 0.010 network), AD (0.727 in the *p* < 0.050; 0.754 in the *p* < 0.010 network), BLAa (0.716 in the *p* < 0.050; 1.000 in the *p* < 0.010 network), BLAp (0.727 in the *p* < 0.050; 0.905 in the *p* < 0.010 network), BMEa (0.696 in the *p* < 0.050; 0.906 in the *p* < 0.010 network), BMEp (0.686 in the *p* < 0.050; 0.692 in the *p* < 0.010 network), LAd (0.706 in the *p* < 0.050; 0.834 in the *p* < 0.010 network), LAv (0.686 in the *p* < 0.050; 0.929 in the *p* < 0.010 network), and vCA3 (0.686 in the *p* < 0.050; 0.763 in the *p* < 0.010 network), in addition to the RE (0.727 in the *p* < 0.050; 0.651 in the *p* < 0.010 network), were stable nodes with higher Clo in the CFC-5s functional networks (Fig. 5D).

The VO (0.676 in the *p* < 0.050; 0.565 in the *p* < 0.010 network), AD (0.774 in the *p* < 0.050; 0.608 in the *p* < 0.010 network), RSDa (0.762 in the *p* < 0.050; 0.552 in the *p* < 0.010 network), and ECT (0.706 in the *p* < 0.050; 0.565 in the *p* < 0.010 network) were stables nodes with higher Clo in the CFC functional networks (Supplementary Fig. S2).

Overall, converging brain regions had higher centralities of Wdg, Evc, and Clo, whereas half of the brain regions with higher Bet measures also had PC higher than 1.5 in the CFC-5s functional networks (Fig. 4).

**Figure 6:**
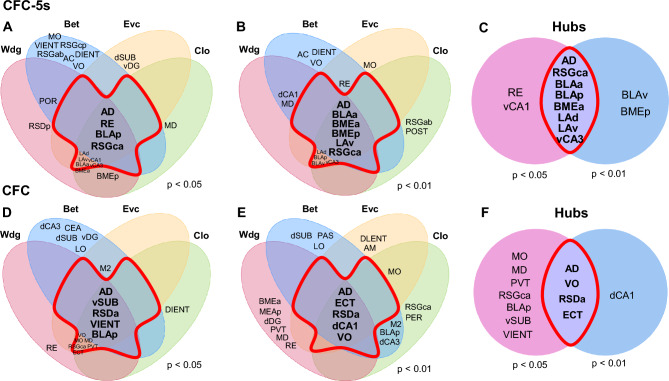
Identifying stable hubs based on centrality measures in the CFC and CFC-5s functional networks. The intersection of the upper 25% of most central regions in the centrality measures of weighted degree (**Wdg**), eigenvector (**Evc**), betweenness (**Bet**), and closeness (**Clo**) in the CFC-5s network using *p* < 0.05 (**A**) or *p* < 0.01 (**B**) thresholds, and in the CFC functional network using (*p* < 0.05) (**D**) or (*p* < 0.01) (**E**)**.** Brain regions in the upper 25% in three or more centrality measures were considered hubs. They are shown inside the red perimeter. Hubs of each threshold level were then intersected to identify stable hubs across the threshold levels (*p* < 0.05 and *p* < 0.01) in the CFC-5s (**C**) and the CFC (**F**) functional networks. Stable hubs were shown inside the red perimeter. See group names in Fig. 1.

Brain regions in the upper 25% in 3 or more centrality measures were considered hubs^30,31^. One subdivision of the RSC (RSGca), one nucleus of the thalamus (AD), five amygdala nuclei (the BLAa, BLAp, BMEa, LAd, and LAv), and one subdivision of the VH (vCA3) were stable hubs in the CFC-5s functional networks (Fig. 6A–C). In turn, the VO, RSDa, AD, and ECT were stable hubs of the CFC functional networks (Fig. 6D–F). The AD was the only common hub of the CFC and CFC-5s networks.

Overall, these results indicated a central role of amygdala nuclei, especially from the basolateral complex (subdivisions of the BLA, BME, and LA), in the CFC-5s functional networks, which is consistent with the previous findings showing higher connectivity of the RSC, VH, and amygdala nuclei (Fig. 2A), and increased internal amygdala connectivity, with a strengthening of LAd-BLAa and LAv-BMEa connections (Fig. 2D) in the CFC-5s functional networks.

### The BLA has higher centralities in the CFC-5s than in the CFC functional networks

Permutation tests directly compared centrality measures between the CFC-5s and CFC functional networks. We calculated the centrality differences between CFC and CFC-5s functional networks for each centrality measure in each brain region. The p-value was expressed as the frequency that the resampling difference (obtained from resampled networks, in which the animal's groups were randomized, without replacement) was higher than the observed difference^31^.

The BLAa (Evc *p* = 0.001 in the *p* < 0.050; *p* = 0.050 in the *p* < 0.010 network; Clo *p* = 0.050 in the *p* < 0.010 network) and BLAv (Evc *p* = 0.050 in *p* < 0.010 and *p* < 0.050 networks) had stable higher centralities in the CFC-5s compared to the CFC functional networks. The POR (Wdg *p* = 0.001 in the *p* < 0.050 and *p* < 0.010 networks; Evc *p* = 0.001 and Clo *p* = 0.050 in the *p* < 0.050 network) had stable higher centralities in the CFC compared to the CFC-5s functional networks (Supplementary Table S3). Although not stable across the thresholds levels, the AC (Bet *p* = 0.001), IL (Wdg *p* = 0.050; Evc *p* = 0.001), DLO (Evc *p* = 0.050), RSDp (Evc *p* = 0.001), BMEp (Wdg and Evc *p* = 0.050), and vCA1 (Wdg, Evc, and Clo *p* = 0.001) had higher centralities in the CFC-5s functional network using *p* < 0.050 and the RSGab (Clo *p* = 0.050), MD (Bet *p* = 0.001), and LAv (Wdg *p* = 0.050; Evc and Clo *p* = 0.001) higher centralities in the CFC-5s functional network using *p* < 0.01. Besides the higher centrality, the MD, BMEp, LAv, and vCA1 were also hubs in their CFC-5s functional networks. In turn, the RSDa (Wdg and Evc *p* = 0.001), dSUB (Bet *p* = 0.001), PAS (Wdg *p* = 0.050; Evc and Clo *p* = 0.001), DIENT (Evc and Clo *p* = 0.050), PER (Bet *p* = 0.050), and VIENT (Wdg and Evc *p* = 0.050) had higher centralities in the CFC functional network using *p* < 0.050, and the PVT (Wdg and Evc *p* = 0.050), and MEAp (Wdg *p* = 0.050) in the CFC functional network using *p* < 0.010. The RSDa, MEAp, and dSUB were also hubs in their CFC functional networks. We summarized all results in Table 2, fully described in Supplementary Table S3. Overall, besides being a hub in the CFC-5s functional networks and having a stronger connection to the LAd, the BLAa had higher centrality in the CFC-5s than in the CFC functional networks, consistently relevant for the CFC-5s networks.

**Table 2:**
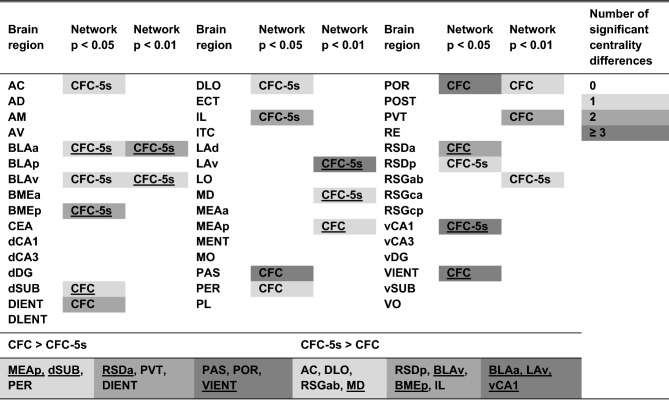
Summary of the centrality comparisons between the CFC and CFC-5s functional networks.

Shades of grey (scale, right) represent the number of centrality measures in which the brain region had higher centrality in the group comparisons in each threshold level (*p* < 0.05 and *p* < 0.01). Group's name represents which one had the significantly higher centrality. Brain regions previously identified as hubs had the group's name subscripted. The lower table summarizes the brain regions with higher centrality measures in the CFC and CFC-5s groups, grouped by the number of significantly higher centrality measures (scale, right), irrespective of threshold levels. Brain regions previously identified as hubs were subscripted. Brain region names are described in Table 1. See group names in Fig. 1. See the p-values of the comparison of centrality measures between the CFC-5s and CFC networks in Supplementary Table S3.

## Discussion

By populational and network evidence, we investigated the activity and interactivity of brain regions underlying a contextual and temporal association. The activity of individual brain regions showed that the CFC-5s specifically activated the PL, IL, BLAp, LAv, MEAa, vCA1, vSUB, PER, POR, DIENT, and VIENT. Evidence from functional connectivity showed that the amygdala nuclei strengthened external connectivity with the RSC, DH, VH, and thalamic nuclei and internal connectivity with stronger LAd-BLAa and LAv-BMEa connections. Network evidence showed that the RSGca, AD, amygdala nuclei (BLAa, BLAp, BMEa, LAd, and LAv), and vCA3 were hubs based on centrality; the MD and RE were connector hubs, and the BLAa and BLAv had higher centralities in the CFC-5s than the CFC network. Thereby, findings indicate increased activity of the mPFC and rhinal cortices at the individual level, increased interactivity of thalamic nuclei and RSC at the network level, and increased activity and interactivity of amygdala nuclei and VH, at both individual and network levels, in temporal associations.

These results are in line with pharmacological and electrophysiology studies showing that the PL^32,33^, IL^34,35^, BLA^32,36^, LA^11,37^, vCA1^38,39^, LEC^12,40^, PER^13,41^, and POR^9^ are engaged in trace conditioning and the PL^42^, IL^43^, VH^44,45^, EC^46,47^, PER^48^, and POR^49^ in SWM. As in CFC-5s, these learnings have time intervals between stimuli or stimuli and actions/outcomes^1–4^. Our results strengthen the interpretation that these brain regions can support transient memory of stimuli and their association, and not other processes, given that controls addressed contextual learning, non-associative learning, and overlapped fear associations. We expanded their role in transient memory of a contextual stimulus, provided observational evidence from immediate early gene (IEG) expression, and dissected brain regions to reveal contributions from more specific subdivisions.

The CFC-5s is a protocol for studying stimuli maintenance and association that has rapid and well-delimited learning. It also requires one pairing to elicit CR and thus would not recruit US expectancy, tracking timing, CS retrieval, action plans, motor selection, or inhibitory control, processes related to multiple pairings^50,51^. Unlike trace conditioning, which can have second-order conditioning by context-US and CS-context associations, the context is already the CS in the CFC-5s training, and there are no other salient stimuli^52^. We have previously behaviorally standardized all groups^22^. The CFC, CFC-5s, CT, CT-5s, and US groups in this Experiment were performed in identical experimental design and conditions of the present study and close in time. Animals were the same age; breeder and cared at the same conditions and had the same housing; the conditioning chamber, experimental room, experiment time, freezing score, and investigator were also the same. CFC-5s elicited specific CR to the conditioned context, similar to the CFC group, and higher freezing responses than the US, CT, and CT-5s unconditioned groups^22^.

We hypothesized that CFC-5s is learned by associating the past context of the animal with the US, given that there is not enough time to recognize the current one, and there are no other salient cues. This past context could be incidentally maintained during a short time interval or having its retrieval facilitated by the temporal proximity with the aversive event. In this view, CFC-5s would additionally engage brain regions forming contextual representations that have functional connections with brain regions that transiently maintain it, which would functionally connect with brain regions that associate the transient CS memory with the US.

To our knowledge, it is the first time temporal associations have been investigated at the network level. Given that only a subtle interval differs the CFC-5s from CFC training, its underlying mechanisms could be better reflected in how brain regions are functionally connected and organized at the network level rather than individual engagement. In addition, memory conceptualizations propose that coordinated activity in distributed brain regions supports memory formation rather than discrete brain regions^7^.

The amygdala nuclei, especially the BLAa, were the most critical nodes in the CFC-5s network. In line, the CFC-5s training activated three amygdala nuclei more than the CFC, in addition to the BLAa and CEA activated in both fear conditioning. Indeed, the pretraining lesions of the BLAp did not impair the CFC^53^. As in the CFC-5s, the contextual learning separated 24 h from the CFC activated more the LAv and MEA than the CFC alone^54^. The pretraining lesion or inactivation of the BLAa or CEA impaired the CFC^53,55^. Because the PL projects to the BLAa^56^, an additional engagement of the PL-BLAa connection could underly the greater relevance of BLAa in the CFC-5s network. The PL could convey to the BLAa past contextual states to be associated at the time of the US delivery, given that there are no other salient stimuli nor time to identify the context. The neural representation of the CS, supported by distinct brain regions and other potential predictors, could converge in the amygdala nuclei to be associated with the US through different and multiple pathways, activating spreader amygdala nuclei than in CFC. In line, projections from the PL to the BLA were required for learning trace conditioning but not CFC^34,57,58^.

The RE and MD were connector hubs in the CFC-5s networks, which agrees with the observations that the thalamus interconnects cortical and subcortical areas, relaying and integrating sensory information^59^. The MD and RE are well-positioned to support PL functions and were engaged in SWM^60,61^ and trace conditioning^62,63^.

The PL has sustained firing during time intervals^14^; activity related to specific contexts^64^; IEG induced by contextual learning^65^, and spatially tuning neurons^60,66^, although it is not required for CFC training^21^, features compatible with a role in maintaining contextual representation over time in the CFC-5s training. The PL could also support other functions based on other mPFC-dependent tasks, such as reducing the interference of the time interval or previous context-safety associations^67^, monitoring the behavioral relevance of stimuli^68^, or mediating the integration of time and space elements in unified memories^69^.

The PL was not a hub or had higher centralities in CFC-5s networks. On the contrary, the PL had the lowest and was only consistently connected with the AC, constituting one community. Because communities may reflect functional modules^26^, the PL and AC may have similar and unique roles in the CFC-5s, resulting in an exclusive community and lower activity correlation with the rest of the network. The PL and AC have CS-evoked activity during trace conditioning^70^, suggesting they could support CS maintenance or attentional processes. In the CFC networks, the PL was connected with the IL, M2, and vCA3 and integrated into the largest community, suggesting that it performs a different function. Thereby, activation, indicated by c-Fos expression, and co-activation, indicated by correlations of c-fos expression, can evaluate distinct but complementary aspects of functionality.

The CFC-5s increased the activation of vCA1. Pretraining functional disconnection between the vCA1 and PL also impaired the CFC-5s but not CFC^22^. The vCA1 contributes to contextual learning^71,72^ and has direct projections to the PL, whereas the dCA1 has not^73^ and thus could convey a contextual representation to the PL during the time interval in the CFC-5s training.

The CFC-5s also increased the activation of the PER and POR. The PER^10^, LA^8^, and LEC^9^ have endogenous persistent-firing neurons, which continue to discharge in a self-sustained manner. CS transient memory could be supported by the collective activity of these brain regions, which are reciprocally interconnected^74^. The PER can bind separated stimuli, as shown in fear conditioning using discontinuous tones^13^, and thus could collaborate to bridge the gap through persistent firing or convey contextual inputs from the DH to PL^75^, which are not directly connected^73^. In turn, the POR could monitor the contextual changes in the CFC-5s. POR neurons are activated when cues are moved in a spatial learning task^76^ and are required for stimulus-driven attention^77^.

The CFC-5s induced activation of more brain regions than CFC, favoring the interpretation that temporal associations are more cognitively demanding^78^. Both conditionings activated the AC, BLAa, CEA, M2, vDG, and RSD, which agree with studies showing that the AC^79,80^, the RSC^81^, and the VH^71^ are engaged in CFC learning. The VO, RSDa, AD, and ECT were stable hubs in the CFC networks. The AD and RSC are part of the extended hippocampal system loop, the Papez circuit, associated with hippocampal-related functions^82^. The ECT is an association area that receives mostly unimodal visual information^75^, which is required for CFC learning^8,13^. CFC networks also had communities with an over-representation of the hippocampus and the amygdala nuclei, which could reflect functional modules related to contextual learning and the context-US association, respectively^16,83^.

This accordance with CFC neurobiology is relevant, given that we showed observational and correlational evidence. Causal evidence between brain regions' activity and learning-related behavior from loss-of-function and gain-of-function manipulations would further confirm the findings from the network-based analysis. Previous studies have characterized the functional network underlying the retrieval of recent and remote contextual fear memories^84,85^ and CFC learning without the DH^25^. We contributed by characterizing the functional network engaged in CFC learning. Findings from the c-Fos correlation between brain regions have been previously validated by chemogenetic manipulation of hubs, indicating a predictive value of graph analysis and convergence from observational and causal approaches^85^. Here, amygdala nuclei had increased importance across different network analyses, indicating internal consistence.

The CFC-5s has a similar experimental design to the contextual pre-exposure facilitation effect (CPFE) paradigm, with the difference that the latter has a 24-h interval. Long and short intervals can lead to different learning mechanisms. In the CPFE, the context is consolidated and retrieved from long-term memory by pattern completion by a brief contextual re-exposure before the US or other cues previously associated with it. The context and the context-US association are encoded in different sessions, better allowing their study separately. The context is also encoded as a configural representation rather than an elemental^86^. In the CFC-5s, we hypothesized that the context is maintained by a transient memory or retrieved by short-term memory, and the context and the context-US association are encoded in a single session. Therefore, the CFC-5s is a suitable model to study transient contextual memories and their fear association, and the CPFE consolidated contextual representations and their fear association.

Contextual pre-exposure increases IEGs in the PL^65^, and the blockage of NMDAR in the PL before the pre-exposure impaired the CPFE^87^. Here, the CFC-5s group had greater c-Fos expression than the CT and CT-5s groups, suggesting that PL could be engaged in additional functions besides contextual learning. Inhibition of the posterior or anterior RSC during or after the re-exposure phase impaired the CPFE^88^. In agreement, we observed higher c-Fos expression in the CFC than the HC groups both in the RSDa and RSDp. The retrieval of the CFC preferentially reactivated CA1 cells activated by the contextual pre-exposure in the CPFE suggesting that CA1 cells encode the fear-associated context^89^. Inactivation of DH before or after, or protein synthesis inhibition in the DH after the pre-exposure, also impaired the CPFE^90–92^. Contextual pre-exposure also increased IEGs in the CA1^65,93^. We did not detect an increase in c-Fos expression in the dCA1, similar to other studies^94,95^. One possibility is that sparse but specific neurons encode contextual memories to discriminate between memories, generating non-overlapping activity patterns^96^.

In short, the functional network of a temporal association had increased amygdala centrality and internal and external connectivity with the RSC, thalamic nuclei, and hippocampus. Amygdala and thalamic nuclei were hubs. The temporal learning also activated eleven brain regions, including amygdala nuclei, VH, and subdivisions of the mPFC, rhinal, and parahippocampal cortices. Together, this system could support transient memories and their fear association.

## Materials and methods

### Subjects

Experiments were conducted on sixty-nine naïve male *Wistar* rats of 10 weeks old weighing 250 to 330 g at the onset of the Experiments. Rats were housed in groups of 4 animals per cage and obtained from CEDEME (Center of Development of Experimental Models for Medicine and Biology, Universidade Federal de São Paulo, Brazil). All animals were acclimatized to their facilities, consisting of transparent polysulfone plastic cages (44 × 35 × 20 cm) individually ventilated, with corn-cob bedding on the floor, for one week before the onset of the Experiments. Room temperature was controlled (22 ± 1 °C), and a light–dark cycle was maintained on a 12 h on–off cycle (lights on at 07:00 am). All experiments were conducted during the light phase of the cycle. Food and water were available ad libitum*,* and animal welfare was assessed daily. The Ethical Committee for Animal Research of Universidade Federal de São Paulo approved the study (number #6790140616). All procedures followed the policies and guidelines of the National Council for the Control of Animal Experimentation (CONCEA, Brazil). The sample size was estimated using the effect size, observed power, and significance level on G*Power^97^ to ensure adequate power to detect effect sizes previously observed using the CFC-5s training^21,22^. The study was conducted in compliance with ARRIVE guidelines^98^.

### Apparatus

The conditioning chamber consisted of a 22 × 27 × 45 cm box with black acrylic walls, a clear acrylic top, and a video camera attached to the top (AVS Projetos, São Paulo, BR). The shock grid comprised parallel stainless-steel bars of 0.4 cm diameter spaced 1.2 cm apart and connected to an electric generator. We cleaned the conditioning chamber with a 20% ethanol solution after the training session for each animal. The transport cage consisted of a 22 × 35 × 20 cm cage with corn-cob bedding from the homecage of the rat and a clear polysulfone cover topped with a flannel. It transported the animals from the homecage to the experimental room, and it was the context during the 5-s interval in the CFC-5s training.

### Behavioral procedures

#### Habituation

Habituations were conducted in the experimental room for three consecutive days before the training sessions. We handled each rat for 5 min in two transport cages, moving them from one to another every 15 s. We performed habituation sessions to familiarize the animals with the experimental conditions, including the transport between the homecage and the experimental room and the contexts of the transport cage and the experimental room. We aimed to decrease the possibility of these contexts being associated with the US in the training session or with the conditioning chamber, functioning as retrieval cues or as the CS in second-order conditioning^22^.

#### Contextual fear conditioning with a 5-s interval (CFC-5s)

The CFC-5s training was conducted as described^21,22^. Rats were pre-exposed to the conditioning chamber for 5 min. Next, we placed them in the transport cage beside the conditioning chamber for a 5-s interval, then re-exposed them to the conditioning chamber, delivering one immediate footshock (0.8 mA, 1 s). The time to remove and return the animals to/from the conditioning chamber was kept to the minimum and took some additional seconds than the 5-s interval but was similar and standardized to all animals. We returned them immediately to their homecage in the transport cage. It has been shown that an immediate footshock is not sufficient to elicit CR or contextual retrieval^86^.

#### Contextual fear conditioning (CFC)

The CFC training was performed as described^21,22^. We placed the rats in the conditioning chamber for 5 min delivering one footshock (0.8 mA, 1 s) at the end. We removed the animals immediately and returned them to their homecage in the transport cage.

### c-Fos immunohistochemistry

Ninety minutes after the training sessions, we anesthetized the rats with IP injections of Lidocaine (10 mg/kg; Bravet, Rio de Janeiro, Brazil) and Thiopental (150 mg/kg; Cristália). Next, we transcardially perfused them with 0.9% saline at 4 °C for 1 min, followed by 4% PFA (paraformaldehyde; Synth, Diadema, Brazil) at 4 °C for 15 min at 12 ml/min. A peristaltic pump (Cole Parmer, Vernon Hills, US) drove the perfusions. We injected 0.1 ml of heparin (Cristália) directly into the left ventricle. We removed the brains from the skull, post-fixed them in 4% PFA for 24 h at 4 °C and transferred them to 30% sucrose (Synth) in 0.02 M KPBS (potassium phosphate-buffered saline) solution until the samples sank (48 h). We freeze the brains with dry ice and store them at -80 °C. We obtained coronal sections of 30 μm in five consecutive series using a cryostat at -20 °C (Leica CM1850, Wetzlar, Germany). We collected all sections between the first (DLO, A*p* + 5.16 mm from bregma) and last (POR, AP -7.80 mm) brain region of interest, and thus each series contained a brain section from DLO to POR every 150 μm. We stored the series in an anti-freezing solution with ethylene glycol (Synth) and 30% sucrose at -20 °C.

Free-floating sections of one series were randomly chosen for each animal and washed in 0.02 M KPBS 3 times for 10 min each. Next, sections were transferred to a solution containing 1% hydrogen peroxide for 15 min at room temperature, rewashed, and transferred to a blocking solution containing 2% normal goat serum (NGS, S-1000–20, Vector Laboratories, Burlingame, US) for 1 h at room temperature. Sections were incubated in the primary antibody solution containing a rabbit anti-c-Fos antibody (1:4000; ab190289, Abcam, Cambridge, UK), 2% NGS, and 0.3% Triton X-100 (85,111, ThermoFisher) for 48 h at 4 °C. Sections were washed and transferred to a secondary antibody solution containing goat anti-rabbit antibody (1:200, BA-1000, Vector Laboratories) and 0.3% Triton X-100 for 90 min at room temperature. Sections were washed and transferred to an avidin–biotin-complex solution (PK-6100, Vector Laboratories) for 90 min at room temperature. Finally, we incubated the sections with DAB (3,3'-Diaminobenzidine, SK-4100, Vector Laboratories) and nickel ammonium sulfate (NAS) for 5 min at room temperature. We mounted the sections on gel-coated slides, dehydrated them with a graded ethanol series of washes, followed by diaphanization in xylol, and coverslipped them using DPX mounting medium (06,522, Sigma–Aldrich). We selected one animal from each group for each batch of immunohistochemistry reaction (6 animals/batch). We performed 12 batches of immunohistochemistry reactions (72 animals).

### Image analysis

We imaged sections on a fluorescent microscope (Olympus, BX50, Waltham, US) outfitted with filters for different excitation/emission wavelengths. We obtained all images at 20 × magnification following the same frame size (4080 × 3072 pixels), image size (0.87 mm × 0.66 mm), and area (0.5742 mm^2^). Brain regions smaller than the standard area were delimited using the ImageJ software (NIH, Washington, United States), excluding adjacent areas from the cell counting. We obtained more extensive brain regions than the standard area in more than one frame, collecting adjacent frames. We took reference images at 4 × magnification to assist in delimitating the brain regions investigated at 20 × magnification. An experimenter, blind to the experimental group of the animals, captured 6 to 8 images from the forty-nine brain regions described in Table 1, from 3 to 4 brain sections of different anteroposterior coordinates (one anterior, one or two intermediate, and one posterior) of both hemispheres, according to neuroanatomical studies^99–102^. We took 6 images (3 bilateral images) from 3 brain sections of the DLO (Anteroposterior, AP, + 5.16 and one brain section + 4.68 mm from bregma); AC, IL, PL (A*p* + 3.72, + 3.00, + 2.76 mm); LO, M2, MO, VO (A*p* + 3.00, + 3.24, + 2.76 mm); AD, AM, AV, MD, PVT, RE, RSDa, RSGca (− 1.80, − 1.92, − 2.04 mm); BLAa, BLAv, BMEa, CEA, LAd, MEAa (AP − 2.28, − 2.64, − 3.00 mm); ITC (AP − 2.00, 02.28, − 3.00 mm); BLAp, BMEp, LAv, MEAp (AP − 2.64, − 3.00, − 3.36 mm); ECT, PER, vCA1, vCA3, vDG, vSUB (AP − 5.40, − 5.64, − 5.88 mm); dSUB, RSDp, RSGab, RSGcp (AP − 5.76, − 6.00, − 6.20 mm); CENT, DIENT, DLENT, MENT, PAS, PRES, POST, VIENT (AP − 6.96, − 7.20, − 7.44 mm); POR (AP − 7.44, − 7.68, − 7.80 mm), and 8 images (4 bilateral images), from 4 brain sections, of the dCA1 (AP − 2.64, − 3.12, − 3.60, − 4.08 mm). Therefore, we had 6 to 8 distinct images (from one or more frames) of a given brain region, from 3 to 4 different brain sections. We counted images from 11 animals/brains in the CFC group; 11 animals/brains in the CFC-5s group; 12 animals/brains in the CT group, 12 animals/brains in the CT-5s group, 11 animals/brains in the US group and 12 animals/brains in the HC group. Brain region abbreviations are described in Table 1. Coordinates were consistent across all animals. We counted the number of c-Fos-positive cells using the CellProfiler software^103^, blinded to the experimental groups. We averaged the counts for each animal (6–8 images) in each brain region and standardized them by the mean expression of the homecage group.

### Experimental design

We map the activity of forty-nine brain regions to determine the set specifically engaged in contextual and temporal fear memories. Brain regions were chosen based on the neurobiology of fear conditioning and relevant PL afferences, given that the PL is required for associations separated in time, such as trace conditioning or CFC-5s, but not overlapped in time, such as the CFC, besides having persistent activity during time intervals that is required for learning^14,15,22^. In addition, we included other brain regions necessary for temporal learning as trace conditioning, such as the AC^78^, IL^34^, PL^15^, RSC^104^, RE^63^, BLA^32^, LA^11^, CEA^105^, DH and VH^39,52^, MEC^47^, LEC^40^, and PER^13^. We also considered their subdivisions since each could have different functions due to distinct projections. We also included brain regions related to contextual learning and CFC that were well-positioned to converge information from the DH to the mPFC, such as the VH, PER, RSC, EC, and thalamic nuclei^10,47,81,84^, given that there are no direct projections between the DH and mPFC^73^. The DH can form contextual representations^83^, and the mPFC to maintain information over time^15^, processes that the CFC-5s learning may involve. Activity was inferred by c-Fos expression, an IEG^106^. We used the CFC-5s task, in which the contextual CS and US are separated in time, and compared to the standard CFC, in which the CS and US are overlapped in time. We also computed the co-activation of brain regions to build functional networks supporting associations of stimuli overlapped and separated in time.

For this, twenty-two male Wistar rats were habituated and trained in the CFC (n = 11) or CFC-5s (n = 11). To better identify brain regions supporting temporal associations, we performed control groups for contextual and non-associative learning, the time interval, and the housing, transport, and handling across the Experiment. The control group for non-associative learning (US group, n = 12) received one immediate footshock in the conditioning chamber (0.8 mA, 1 s). The control group for contextual learning was exposed to the conditioning chamber for 5 min without receiving the footshock (CT group, n = 11). The control group for the time interval was exposed to the conditioning chamber for 5 min, removed by 5s, and then re-placed in it without receiving the footshock (CT-5s group, n = 12). Thus, the CT-5s group was a control for the contextual learning and any processes associated with inserting the time interval, including the interference of being picked up and transferred (Fig. 1G).

Freezing time in the conditioning chamber before the US during the training session was evaluated manually with a stopwatch by an experienced observer and used to measure basal freezing responses to determine if groups were similar a priori. Each homecage had rats from distinct groups. All habituation and training sessions were performed from 10:00 am to 2:00 pm and followed an order to allocate the groups evenly throughout the period. We also distributed the order of removing the animals from their homecage (first to fourth) uniformly in the groups, i.e., each group had at least one animal removed in the first to fourth order from the homecage. All animals, including rats in the homecage during the training session (HC group, n = 12), were euthanized 90 min after the training session for c-Fos immunohistochemistry (Fig. 1F). c-Fos expression 90 min post-training is specific to contextual fear learning in the hippocampus, amygdala nuclei, and cortex^106^. Brain tissue containing more posterior brain regions (like the POR) was lost during cryostat section or cryopreservation in one CFC, CFC-5s, and CT animal. For this reason, these groups had a sample size of 11 animals, and CT-5s, US, and HC groups of 12 animals.

### Statistical analysis

Data were analyzed by Generalized Linear Models (GZLM), a generalization of General Linear Models used to fit regression models for univariate data presumed to follow the exponential class of distributions^107^. Estimations were adjusted to Linear, Gamma, or Tweedie probability distributions according to the Akaike Information Criterion (AIC). We reported the values of the Wald test (W), the degree of freedom (df), and p-values (p). GZLM evaluated the main effect of the group in the freezing time in the training session and the c-Fos-positive cells in each of the forty-nine brain regions investigated. We suggested brain regions specifically engaged in the CFC-5s for those with higher c-Fos expression than all the other groups. We considered brain regions involved in both fear conditioning when CFC and CFC-5s groups, firstly, did not differ; secondly, they had higher c-fos expression than US or HC groups; and thirdly, they had higher c-fos expression than their respectively CT and CT-5s control groups or if CT and CT-5s groups did not differ from the US and HC groups. Estimations were considered statistically significant if *p* < 0.050. In these cases, we used the LSD test when necessary (IBM SPSS Statistics, 23). We also compared effect sizes using standardized regression coefficients (β). Values above 0.35 are considered large^108^. We created all graphs in GraphPad Prism 8 (GraphPad, San Diego, US).

### Correlation matrices

We computed the correlation coefficients (Pearson's r) for the c-Fos expression between all pairs of brain regions in the CFC and CFC-5s groups, generating correlation matrices (IBM SPSS Statistics, 23). Correlation coefficients indicate how well the mean c-Fos expression in one brain region correlates with the mean c-Fos expression in other brain regions, identifying brain regions where c-Fos expression co-varied across the animals. It is assumed that brain regions that change their activity together can be functionally connected^109^.

We next categorized the forty-nine brain regions into nine anatomical groups to reflect major brain subdivisions (Table 1). We computed the mean correlation coefficients within the same anatomical group (internal connectivity), between one anatomical group and all the other ones (external connectivity), or between pairs of anatomical groups and used the GZLM to evaluate the main effect of the group in the mean of the correlation coefficients^85^. Estimations were considered statistically significant if *p* < 0.050. We also computed the standardized differences of correlation coefficients to compare the CFC and CFC-5s groups. We normalized the correlation coefficients using Fisher's Z transformation and calculated the difference between the CFC and CFC-5s groups (IBM SPSS Statistics, 23). Standardized differences (Fisher's Z differences) ≥|2| were considered to correspond to a level of significance of α = 0.05^110^. We next investigated factors that may influence the detection of correlations. To verify whether the functional connectivity was determined by the magnitude of the activity in each brain region, we performed Linear Regressions to observe the proportion of the strengths of the correlation coefficients explained by the c-Fos expression in each brain region. We next computed the correlations between the mean r and the mean c-Fos expression and between the mean r^2^ and the mean c-Fos expression of all brain regions. Models were considered statistically significant if *p* < 0.050 (IBM SPSS Statistics, 23).

### Functional networks

We used the correlation matrices to generate functional networks for the CFC and CFC-5s groups. We selected the significant correlation coefficients using two p-values (*p* < 0.050 or *p* < 0.010) for each group, one more and the other less conservative. Thus, we generated two functional networks, one for each p-value, and used them to identify stable properties in the CFC and CFC-5s functional networks, regardless of the threshold used to build them^25^. The functional networks reflected statistical rather than neuroanatomical connections^24^. They consisted of nodes (brain regions) connected by undirected edges (connections) representing correlation coefficients above the threshold level (*p* < 0.050 and *p* < 0.010). GZLM evaluated the main effect of the group (CFC or CFC-5s) or threshold of p-values (*p* < 0.050 or *p* < 0.010) in the average degree, average weighted degree, global efficiency, nodal efficiency, and average cluster coefficient. Two-samples Kolmogorov–Smirnov (K-S) tests compared the distributions of the same measures. Estimations were considered statistically significant if *p* < 0.050. The average degree was computed as the average number of edges (functional connections) per node (brain region), and the average weighted degree as the average degree pondered by the weight of the correlation coefficient. The global efficiency was calculated as the network average of the nodal efficiencies of all nodes (computed for each node as the inverse of the harmonic mean of the shortest path length, the minimum number of edges, between the node and all the others), and includes disconnected nodes, and the local efficiency as the network average of the nodal efficiencies of the neighbors of a node, excluding the node itself^24^. The average cluster coefficient was computed as the network average of the number of neighbors connected from the total number of possible functional connections among the neighbors of a node (i.e., the number of connected triangles from the possible ones)^23^.

#### Graph analysis

All graph analyses were performed in R Studio 4 (R Studio) using custom-written routines developed previously in our research group^31^, which are freely accessible (https://github.com/coelhocao/Brain_Network_analysis), and the packages igraph^111^, matrix^112^, lattice^113^, car^114^, and VennDiagram^115^. We drew or adapted Figures from R Studio in the Photoshop CS6 software.

#### Edge's measures

We normalized the correlation coefficients using the Fisher's Z transformation and compared the Fisher transformed values (Fisher's Z) distributions of the CFC and CFC-5s networks in each threshold level using a two-sample Kolmogorov–Smirnov test. Tests were considered statistically significant if *p* < 0.050^109^.

#### Community measures

We partitioned the functional networks into sub-units called communities^25^, computed the network modularity (quality of the partition of nodes into communities), and described the nodes of each community and the proportion of anatomical groups in them. We next computed the community measures of WC z-score and PC to identify provincial and connector hubs. Hubs are brain regions (nodes) that occupy a central position in the organization of functional networks^116^. The WC z-scores were computed as the number of edges of a node inside its community, normalized by the community average. The PC was calculated as the ratio between the number of edges of a node inside its community to the total of edges in the network, subtracted from 1^26^. Provincial hubs were brain regions with more connections inside their community (WC z-score ≥ 1.5 and PC ≤ 0.3). Connector hubs were brain regions with a higher proportion of connections outside their community (PC ≥ 0.8 and WC z-score ≤ 1.5)^27^.

#### Node's measures

We analyzed the properties of the nodes by the centrality measures of Wdg, Evc, Bet, and Clo. The Wdg was computed as the sum of the correlation coefficients of a node^28^. The Wdg considers that important brain regions have more and stronger connections. The Evc was computed as the sum of the eigenvalues of the neighboring nodes of a node^29^. The Evc considers that important brain regions are connected to brain regions with more connections, capturing the influence of the number of connections of one neighboring brain region in the others. The Bet was computed as the shortest path between all pairs of nodes that pass through the node^28^. The Bet considers that important brain regions are more connectors and participative, helping to connect unconnected brain regions. The Clo was computed as the average of the shortest paths between the node and all the other nodes^28^. The Clo considers that important brain regions are closer to others.

We ranked the nodes in decrescent order according to their values in each centrality measure to identify hubs based on centrality measures. The upper 25% of nodes in 3 or more centrality measures were considered hubs^116^. Next, we performed a permutation test to directly compare the centralities of the brain regions in the CFC and CFC-5s functional networks^31^. We randomized the group label of each animal without replacement and generated new functional networks for each group (CFC and CFC-5s) and threshold level (*p* < 0.050 and *p* < 0.010). We then computed the centrality measures in these networks and calculated the centrality differences between the CFC and CFC-5s networks. We repeated these steps 1000 times, generating 1000 resampling differences for each centrality measure in each brain region. The p-value was expressed as the frequency that the resampling difference was higher than the observed difference from the empirical networks (*p* = resampling difference > empirical difference / 1000).

### Supplementary Information

Supplementary Information.

## Data Availability

The datasets generated during and/or analyzed during the current study are available from the corresponding author upon reasonable request.

## Accession codes

All codes used are freely accessible at https://github.com/coelhocao/Brain_Network_analysis.

## References

[1] Olton DS (1979). Mazes, maps, and memory. Am. Psychol..

[2] Constantinidis C (2018). Persistent spiking activity underlies working memory. J. Neurosci..

[3] Pavlov I (1927). Conditioned Reflexes: An Investigation of the Physiological Activity of the Cerebral Cortex.

[4] Raybuck JD, Lattal KM (2014). Bridging the interval: Theory and neurobiology of trace conditioning. Behav. Proc..

[5] Semon RW (1923). Mnemic Psychology.

[6] Hebb DO (1949). The Organization of Behavior: A Neuropsychological Theory.

[7] Josselyn SA, Tonegawa S (2020). Memory engrams: Recalling the past and imagining the future. Science.

[8] Navaroli VL, Zhao Y, Boguszewski P, Brown TH (2012). Muscarinic receptor activation enables persistent firing in pyramidal neurons from superficial layers of dorsal Perirhinal cortex. Hippocampus.

[9] Suter EE, Weiss C, Disterhoft JF (2013). Perirhinal and postrhinal, but not lateral entorhinal, cortices are essential for acquisition of trace eyeblink conditioning. Learn. Mem..

[10] Kholodar-Smith DB, Allen TA, Brown TH (2008). Fear conditioning to discontinuous auditory cues requires perirhinal cortical function. Behav. Neurosci..

[11] Baysinger AN, Kent BA, Brown TH (2012). Muscarinic receptors in amygdala control trace fear conditioning. PLoS ONE.

[12] Tanninen SE (2015). Cholinergic, but not Nmda, receptors in the lateral entorhinal cortex mediate acquisition in trace eyeblink conditioning. Hippocampus.

[13] Kholodar-Smith DB, Boguszewski P, Brown TH (2008). Auditory trace fear conditioning requires perirhinal cortex. Neurobiol. Learn. Mem..

[14] Gilmartin MR, McEchron MD (2005). Single neurons in the medial prefrontal cortex of the rat exhibit tonic and phasic coding during trace fear conditioning. Behav. Neurosci..

[15] Gilmartin MR, Miyawaki H, Helmstetter FJ, Diba K (2013). Prefrontal activity links nonoverlapping events in memory. J. Neurosci..

[16] Sun Y, Gooch H, Sah P (2020). Fear conditioning and the basolateral amygdala. F1000Research.

[17] Wolff SB (2014). Amygdala interneuron subtypes control fear learning through disinhibition. Nature.

[18] Gore F (2015). Neural representations of unconditioned stimuli in basolateral amygdala mediate innate and learned responses. Cell.

[19] Barot SK, Kyono Y, Clark EW, Bernstein IL (2008). Visualizing stimulus convergence in amygdala neurons during associative learning. Proc. Natl. Acad. Sci. USA.

[20] Barot SK, Chung A, Kim JJ, Bernstein IL (2009). Functional imaging of stimulus convergence in amygdalar neurons during pavlovian fear conditioning. PLoS ONE.

[21] Santos TB, Kramer-Soares JC, Favaro VM, Oliveira MGM (2017). Involvement of the prelimbic cortex in contextual fear conditioning with temporal and spatial discontinuity. Neurobiol. Learn. Mem..

[22] Santos TB, Wallau AE, Kramer-Soares JC, Oliveira MGM (2020). Functional interaction of ventral hippocampal CA1 region and prelimbic cortex contributes to the encoding of contextual fear association of stimuli separated in time. Neurobiol. Learn. Mem..

[23] Watts DJ, Strogatz SH (1998). Collective dynamics of 'small world' networks. Nature.

[24] Latora V, Marchiori M (2001). Efficient behavior of small-world networks. Phys. Rev. Lett..

[25] Blondel, V. D., Guillaume, J. L., Lambiotte, R., Lefebvre, E. Fast unfolding of communities in large networks. Preprint at https://arxiv.org/abs/0803.0476 (2008).

[26] Guimerà R, Amaral LA (2005). Cartography of complex networks: modules and universal roles. J. Stat. Mech..

[27] Cohen JR, D'Esposito M (2016). The segregation and integration of distinct brain networks and their relationship to cognition. J. Neurosci..

[28] Rubinov M, Sporns O (2010). Complex network measures of brain connectivity uses and interpretations. Neuroimage.

[29] Ruhnau B (2000). Eigenvector-centrality—A node-centrality?. Soc. Netw..

[30] Van den Heuvel MP, Sporns O (2011). Rich-club organization of the human connectome. J. Neurosci..

[31] Coelho CAO, Ferreira TL, Kramer-Soares JC, Sato JR, Oliveira MGM (2018). Network supporting contextual fear learning after dorsal hippocampal damage has increased dependence on retrosplenial cortex. PLOS Comput. Biol..

[32] Guimarãis M, Gregório A, Cruz A, Guyon N, Moita MA (2011). Time determines the neural circuit underlying associative fear learning. Front. Behav. Neurosci..

[33] Gilmartin MR, Kwapis JL, McEchron MD (2012). Trace and contextual fear conditioning are impaired following unilateral microinjection of muscimol in the ventral hippocampus or amygdala, but not the medial prefrontal cortex. Neurobiol. Learn. Mem..

[34] Song C, Ehlers VL, Moyer JR (2015). Trace fear conditioning differentially modulates intrinsic excitability of medial prefrontal cortex-basolateral complex of amygdala projection neurons in infralimbic and prelimbic cortices. J. Neurosci..

[35] Mukherjee A, Caroni P (2018). Infralimbic cortex is required for learning alternatives to prelimbic promoted associations through reciprocal connectivity. Nat. Commun..

[36] Chau LS, Prakapenka A, Fleming SA, Davis AS, Galvez R (2013). Elevated Arc/Arg 3.1 protein expression in the basolateral amygdala following auditory trace-cued fear conditioning. Neurobiol. Learn. Memory.

[37] Kim N, Kong MS, Jo KI, Kim EJ, Choi JS (2015). (2015) Increased tone-offset response in the lateral nucleus of the amygdala underlies trace fear conditioning. Neurobiol. Learn. Mem..

[38] Rogers JL, Hunsaker MR, Kesner RP (2006). Effects of ventral and dorsal Ca1 subregional lesions on trace fear conditioning. Neurobiol. Learn. Mem..

[39] Hudgins C, Otto T (2019). Hippocampal arc protein expression and conditioned fear. Neurobiol. Learn. Mem..

[40] Pilkiw M (2017). Phasic and tonic neuron ensemble codes for stimulus-environment conjunctions in the lateral entorhinal cortex. Elife.

[41] Bang SJ, Brown TH (2009). Muscarinic receptors in perirhinal cortex control trace conditioning. J. Neurosci..

[42] Kim D (2016). Distinct roles of parvalbumin- and somatostatin-expressing interneurons in working memory. Neuron.

[43] Ragozzino ME, Adams S, Kesner RP (1998). Differential involvement of the dorsal anterior cingulate and prelimbic-infralimbic areas of the rodent prefrontal cortex in spatial working memory. Behav. Neurosci..

[44] Wang GW, Cai XJ (2006). Disconnection of the hippocampal-prefrontal cortical circuits impairs spatial working memory performance in rats. Behav. Brain Res..

[45] O'Neill PK, Gordon JA, Sigurdsson T (2013). Theta oscillations in the medial prefrontal cortex are modulated by spatial working memory and synchronize with the hippocampus through its ventral subregion. J. Neurosci..

[46] Ramirez JJ (2007). Bilateral entorhinal cortex lesions impair acquisition of delayed spatial alternation in rats. Neurobiol. Learn. Mem..

[47] Suh J, Rivest AJ, Nakashiba T, Tominaga T, Tonegawa S (2011). Entorhinal cortex layer III input to the hippocampus is crucial for temporal association memory. Science.

[48] Wiig KA, Bilkey DK (1994). The effects of perirhinal cortical lesions on spatial reference memory in the rat. Behav. Brain Res..

[49] Bussey TJ, Muir JL, Aggleton JP (1999). Functionally dissociating aspects of event memory: The effects of combined perirhinal and postrhinal cortex lesions on object and place memory in the rat. J. Neurosci..

[50] Pilkiw M, Takehara-Nishiuchi K (2018). Neural representations of time-linked memory. Neurobiol. Learn. Mem..

[51] Kamigaki T, Dan Y (2017). Delay activity of specific prefrontal interneuron subtypes modulates memory-guided behavior. Nat. Neurosci..

[52] Huerta PT, Sun LD, Wilson MA, Tonegawa S (2000). Formation of temporal memory requires Nmda receptors within Ca1 pyramidal neurons. Neuron.

[53] Goosens KA, Maren S (2001). Contextual and auditory fear conditioning are mediated by the lateral, basal, and central amygdaloid nuclei in rats. Learn. Mem..

[54] Trogrlic L, Wilson YM, Newman AG, Murphy M (2011). Context fear learning specifically activates distinct populations of neurons in amygdala and hypothalamus. Learn. Mem..

[55] Wilensky AE, Schafe GE, Kristensen MP, LeDoux JE (2006). Rethinking the fear circuit: The central nucleus of the amygdala is required for the acquisition, consolidation, and expression of Pavlovian fear conditioning. J. Neurosci..

[56] Mcdonald AJ, Mascagni F, Guo L (1996). Projections of the medial and lateral prefrontal cortices to the amygdala: A phaseolus vulgaris leucoagglutinin study in the rat. Neuroscience.

[57] Kirry AJ, Twining RC, Gilmartin MR (2020). Prelimbic input to basolateral amygdala facilitates the acquisition of trace cued fear memory under weak training conditions. Neurobiol. Learn. Mem..

[58] Kitamura T (2017). Engrams and circuits crucial for systems consolidation of a memory. Science.

[59] Fama R, Sullivan EV (2015). Thalamic structures and associated cognitive functions: Relations with age and aging. Neurosci. Biobehav. Rev..

[60] Bolkan SS (2017). Thalamic projections sustain prefrontal activity during working memory maintenance. Nat. Neurosci..

[61] Maisson DJ, Gemzik ZM, Griffin AL (2018). Optogenetic suppression of the nucleus reuniens selectively impairs encoding during spatial working memory. Neurobiol. Learn. Mem..

[62] Powell DA, Churchwell J (2002). Mediodorsal thalamic lesions impair trace eyeblink conditioning in the rabbit. Learn. Mem..

[63] Eleore L, López-Ramos JC, Guerra-Narbona R, Delgado-García JM (2011). Role of reuniens nucleus projections to the medial prefrontal cortex and to the hippocampal pyramidal Ca1 area in associative learning. PLoS ONE.

[64] Hyman JM, Ma L, Balaguer-Ballester E, Durstewitz D, Seamans JK (2012). Contextual encoding by ensembles of medial prefrontal cortex neurons. Proc. Natl. Acad. Sci. USA.

[65] Asok A, Schreiber WB, Jablonski SA, Rosen JB, Stanton ME (2013). Egr-1 increases in the prefrontal cortex following training in the context preexposure facilitation effect (CPFE) paradigm. Neurobiol. Learn. Mem..

[66] Spellman T (2015). Hippocampal-prefrontal input supports spatial encoding in working memory. Nature.

[67] Guise KG, Shapiro ML (2017). Medial prefrontal cortex reduces memory interference by modifying hippocampal encoding. Neuron.

[68] Jarovi, J., Volle, J., Yu, X., Guan, L., Takehara-Nishiuchi, K. Prefrontal theta oscillations promote selective encoding of behaviorally relevant events. *eNeuro*. **5(6)**, ENEURO.0407-18.2018 (2018). 10.1523/ENEURO.0407-18.2018 PMC6348453 30693310

[69] Preston AR, Eichenbaum H (2013). Interplay of hippocampus and prefrontal cortex in memory. Curr. Biol..

[70] Steenland HW, Li XY, Zhuo M (2012). Predicting aversive events and terminating fear in the mouse anterior cingulate cortex during trace fear conditioning. J. Neurosci..

[71] Rudy JW, Matus-Amat P (2005). The ventral hippocampus supports a memory representation of context and contextual fear conditioning: Implications for a unitary function of the hippocampus. Behav. Neurosci..

[72] Kim WB, Cho JH (2020). Encoding of contextual fear memory in hippocampal-amygdala circuit. Nat. Commun..

[73] Hoover WB, Vertes RP (2007). Anatomical analysis of afferent projections to the medial prefrontal cortex in the rat. Brain Struct. Funct..

[74] Pitkänen A, Pikkarainen M, Nurminen N, Ylinen A (2000). Reciprocal connections between the amygdala and the hippocampal formation, perirhinal cortex, and postrhinal cortex in rat. A review. Ann. N. Y. Acad. Sci..

[75] Furtak SC, Wei SM, Agster KL, Burwell RD (2007). Functional neuroanatomy of the parahippocampal region in the rat: The perirhinal and postrhinal cortices. Hippocampus.

[76] Burwell RD, Hafeman MD (2003). Positional firing properties of postrhinal cortex neurons. Neuroscience.

[77] Trettel, S. G., Agster, K. L., Burwell, R. D. Perirhinal and postrhinal damage have different consequences on attention as assessed in the five-choice serial reaction time task. *eNeuro*. **8(5)**, ENEURO.0210–21.2021 (2021). 10.1523/ENEURO.0210-21.2021 PMC8462067 34475265

[78] Han CJ (2003). Trace but not delay fear conditioning requires attention and the anterior cingulate cortex. Proc. Natl. Acad. Sci. USA.

[79] Vetere G (2011). Spine growth in the anterior cingulate cortex is necessary for the consolidation of contextual fear memory. Proc. Natl. Acad. Sci. USA.

[80] Einarsson EÖ, Nader K (2012). Involvement of the anterior cingulate cortex in formation, consolidation, and reconsolidation of recent and remote contextual fear memory. Learn. Mem..

[81] Keene CS, Bucci DJ (2008). Contributions of the retrosplenial and posterior parietal cortices to cue-specific and contextual fear conditioning. Behav. Neurosci..

[82] Papez JW (1937). A proposed mechanism of emotion. J. Neuropsychiatry Clin. Neurosci..

[83] Fanselow MS (2010). From contextual fear to a dynamic view of memory systems. Trends Cogn. Sci..

[84] Wheeler AL (2013). Identification of a functional connectome for for long-term fear memory in mice. PLoS Comput. Biol..

[85] Vetere G (2017). Chemogenetic interrogation of a brain-wide fear memory network in mice. Neuron.

[86] Fanselow MS (1990). Factors governing one-trial contextual conditioning. Anim. Learn. Behav..

[87] Heroux NA, Horgan CJ, Stanton ME (2021). Prefrontal Nmda-receptor antagonism disrupts encoding or consolidation but not retrieval of incidental context learning. Behav. Brain Res..

[88] Trask S, Helmstetter FJ (2022). Unique roles for the anterior and posterior retrosplenial cortices in encoding and retrieval of memory for context. Cereb Cortex..

[89] Deng W, Mayford M, Gage FH (2013). Selection of distinct populations of dentate granule cells in response to inputs as a mechanism for pattern separation in mice. Elife.

[90] Barrientos RM, O'Reilly RC, Rudy JW (2002). Memory for context is impaired by injecting anisomycin into dorsal hippocampus following context exploration. Behav. Brain Res..

[91] Matus-Amat P, Higgins EA, Barrientos RM, Rudy JW (2004). The role of the dorsal hippocampus in the acquisition and retrieval of context memory representations. J. Neurosci..

[92] Chang S, Liang KC (2012). Roles of hippocampal gabaa and muscarinic receptors in consolidation of context memory and context-shock association in contextual fear conditioning: A double dissociation study. Neurobiol. Learn. Mem..

[93] Heroux NA, Osborne BF, Miller LA, Kawan M, Buban KN, Rosen JB, Stanton ME (2018). Differential expression of the immediate early genes c-Fos, Arc, Egr-1, and Npas4 during long-term memory formation in the context preexposure facilitation effect (CPFE). Neurobiol. Learn. Mem..

[94] Beck CH, Fibiger HC (1995). Conditioned fear-induced changes in behavior and in the expression of the immediate early gene C-Fos: With and without diazepam pretreatment. J. Neurosci..

[95] Chakraborty T, Asok A, Stanton ME, Rosen JB (2016). Variants of contextual fear conditioning induce differential patterns of Egr-1 activity within the young adult prefrontal cortex. Behav. Brain Res..

[96] Park S (2016). Neuronal allocation to a hippocampal engram. Neuropsychopharmacology.

[97] Faul F, Lang A, Buchner A (2007). G*Power 3: A flexible statistical power analysis program for the social, behavioral, and biomedical sciences. Behav. Res. Methods.

[98] Kilkenny C, Browne WJ, Cuthill IC, Emerson M, Altman DG (2010). Improving bioscience research reporting: The ARRIVE guidelines for reporting animal research. PLoS Biol..

[99] Insausti R, Herrero MT, Witter MP (1997). Entorhinal cortex of the rat: Cytoarchitectonic subdivisions and the origin and distribution of cortical efferents. Hippocampus.

[100] Burwell RD (2001). Borders and cytoarchitecture of the perirhinal and postrhinal cortices in the rat. J. Comp. Neurol..

[101] Paxinos G, Watson C (2007). The Rat Brain in Stereotaxic Coordinates.

[102] Sugar J, Witter MP, van Strien NM, Cappaert NL (2011). The retrosplenial cortex: Intrinsic connectivity and connections with the (para)hippocampal region in the rat. An interactive connectome. Front. Neuroinform..

[103] Carpenter AE (2006). Cell profiler: Image analysis software for identifying and quantifying cell phenotypes. Genome Biol..

[104] Kwapis JL, Jarome TJ, Lee JL, Helmstetter FJ (2015). The retrosplenial cortex is involved in the formation of memory for context and trace fear conditioning. Neurobiol. Learn. Mem..

[105] Brande-Eilat N, Golumbic YN, Zaidan H, Gaisler-Salomon I (2015). Acquisition of conditioned fear is followed by region-specific changes in RNA editing of glutamate receptors. Stress..

[106] Stanciu M, Radulovic J, Spiess J (2001). Phosphorylated camp response element binding protein in the mouse brain after fear conditioning: Relationship to FOS production. Mol. Brain Res..

[107] McCullagh P, Nelder JA (1989). Generalized Linear Models.

[108] Cohen JA (1992). Power primer. Psychol. Bull..

[109] Park HJ, Friston K (2013). Structural and functional brain networks: From connections to cognition. Science.

[110] Alstott J, Breakspear M, Hagmann P, Cammoun L, Sporns O (2009). Modeling the impact of lesions in the human brain. PLoS Comput. Biol..

[111] Csardi, G., Nepusz, T. The igraph software package for complex network research: Inter Journal. *Complex Syst*. **1695**; https://igraph.org (2006).

[112] Bates, D., Machler, M. Matrix: Sparse and Dense Matrix Classes and Methods. https://cran.r-project.org/web/packages/Matrix/Matrix.pdf (2022).

[113] Sakar D (2008). Lattice: Multivariate Data Visualization with R.

[114] Fox J, Weisberg S (2011). An R Companion to Applied Regression.

[115] Chen, H. VennDiagram: Generate High-Resolution Venn and Euler Plots https://cran.r-project.org/web/packages/VennDiagram/VennDiagram.pdf (2022).

[116] Van den Heuvel MP, Sporns O (2013). Network hubs in the human brain. Cell.

