# Supplementary material for: Functional network of contextual and temporal memory has increased amygdala centrality and connectivity with the retrosplenial cortex, thalamus, and hippocampus
Source: Sci Rep. 2023 Aug 11;13:13087. doi: 10.1038/s41598-023-39946-1 (PMC10421896; doi:10.1038/s41598-023-39946-1)
Supplement: Supplementary file 1 — Supplementary Information. [file 41598_2023_39946_MOESM1_ESM.pdf]

Functional network of contextual and temporal memory has increased amygdala centrality and connectivity with the retrosplenial cortex, thalamus, and hippocampus

Thays Brenner dos Santos, Juliana Carlota Kramer-Soares, Cesar Augusto de Oliveira Coelho, Maria Gabriela Menezes Oliveira

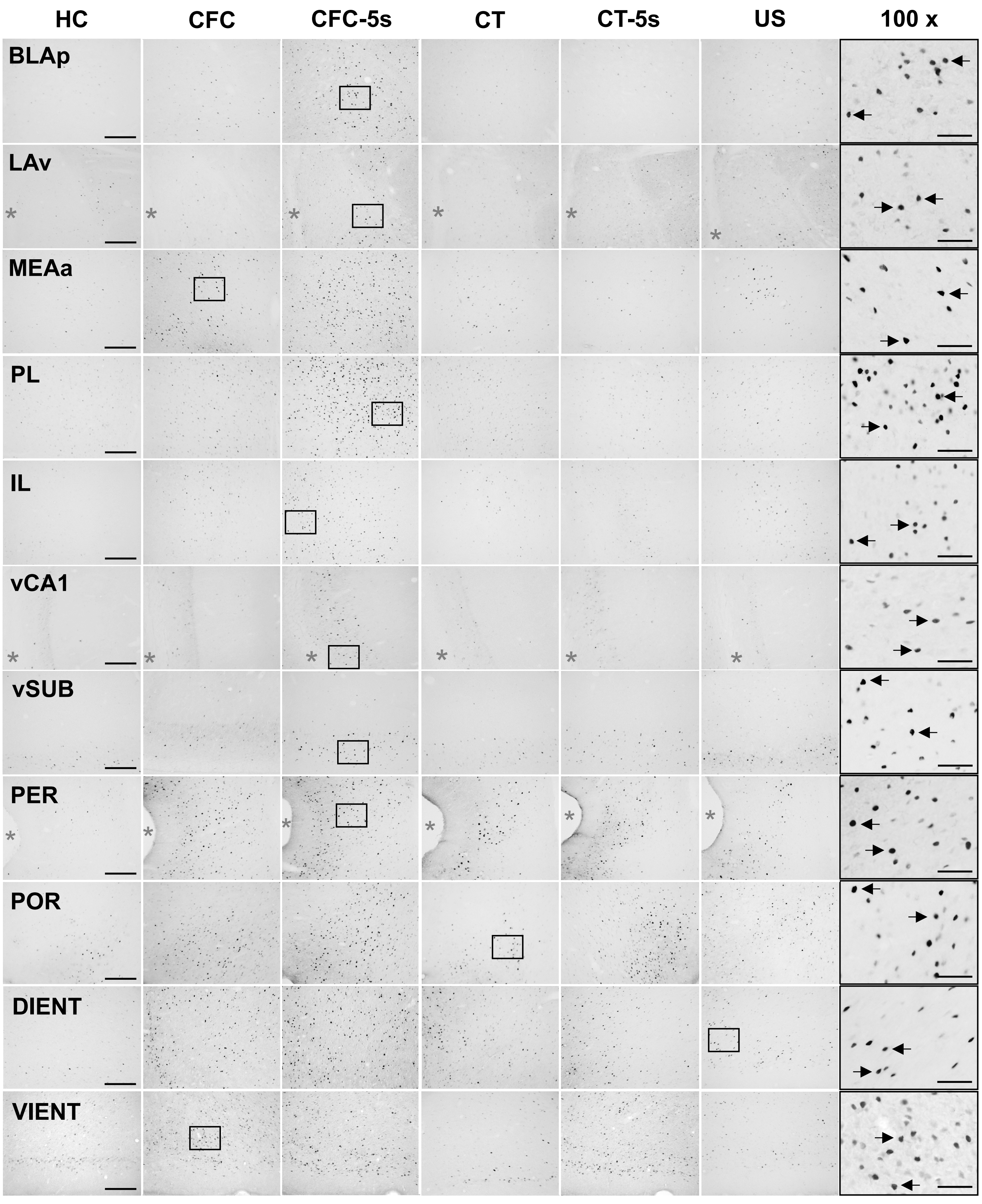
HC
CFC
CFC-5s
CT
CT-5s
US
100 x
BLAp
LAv
MEAa
PL
IL
vCA1
vSUB
PER
POR
DIENT
VIENT

Supplementary Figure S1 – Representative images of c-fos expression induced by HC, CFC, CFC-5s, CT, CT-5s, and US groups. Brain regions with higher c-Fos expression in the CFC-5s group than all the other ones were the basolateral amygdala, posterior (**BLAp**), lateral amygdala, ventral (**LAv**), medial amygdala, anterior (**MEAa**), prelimbic cortex (**PL**), infralimbic cortex (**IL**), ventral CA1 (**vCA1**), ventral subiculum (**vSUB**), perirhinal cortex (**PER**), postrhinal cortex (**POR**), dorsal amygdalar capsule (LAv), or external capsule (vCA1). Black arrows indicate c-Fos-positive cells. Scale bar = 200 μm (left); 50 μm (right).

Supplementary Table S1 – Comparison of mean correlation coefficients within or between major brain subdivisions in the CFC and CFC-5s groups

	CFC (1)	CFC-5s (2)	Wald	P-value	β		CFC (1)	CFC-5s (2)	Wald	P-value	β
**Within**						**Between (inter-regional):**					
**mPFC**	0.733 ± 0.112	0.802 ± 0.097	0.263	0.608	0.280	**mPFC**	**0.403** ± 0.023	**0.284** ± 0.025	**12.581**	**0.001** [1]	**0.404**
**FC**	0.780 ± 0.056	0.664 ± 0.087	1.345	0.246	0.407	**FC**	**0.532** ± 0.018	**0.431** ± 0.018	**15.846**	**0.001** [1]	**0.339**
**RSC**	0.579 ± 0.095	0.693 ± 0.079	0.902	0.342	0.336	**RSC**	**0.450** ± 0.020	**0.517** ± 0.018	**6.340**	**0.012** [2]	**0.226**
**TAL**	0.841 ± 0.053	0.847 ± 0.039	0.007	0.935	0.027	**TAL**	**0.606** ± 0.015	**0.543** ± 0.017	**7.734**	**0.005** [1]	**0.228**
**AMY**	**0.730** ± 0.031	**0.858** ± 0.017	**9.475**	**0.002** [2]	**0.597**	**AMY**	**0.500** ± 0.012	**0.576** ± 0.012	**20.332**	**0.001** [2]	**0.272**
**DH**	0.975 ± 0.011	0.821 ± 0.104	1.836	0.175	0.809	**DH**	**0.588** ± 0.022	**0.480** ± 0.023	**11.840**	**0.001** [1]	**0.393**
**VH**	0.786 ± 0.120	0.889 ± 0.051	0.747	0.387	0.464	**VH**	**0.509** ± 0.021	**0.594** ± 0.021	**8.409**	**0.004** [2]	**0.333**
**SUB**	0.731 ± 0.061	0.624 ± 0.089	1.049	0.306	0.361	**SUB**	0.419 ± 0.019	0.394 ± 0.020	0.806	0.369	0.081
**PHC**	0.828 ± 0.052	0.744 ± 0.064	1.117	0.291	0.318	**PHC**	**0.523** ± 0.019	**0.420** ± 0.021	**23.303**	**0.001** [1]	**0.340**
**Between mPFC and:**						**Between FC and:**					
**FC**	0.566 ± 0.051	0.523 ± 0.061	0.314	0.575	0.200	**RSC**	0.377 ± 0.063	0.446 ± 0.055	0.713	0.399	0.235
**RSC**	0.344 ± 0.081	0.202 ± 0.075	1.765	0.184	0.463	**TAL**	**0.701** ± 0.031	**0.503** ± 0.043	**14.306**	**0.001** [1]	**0.870**
**TAL**	**0.586** ± 0.035	**0.273** ± 0.035	**25.539**	**0.001** [1]	**1.270**	**AMY**	0.579 ± 0.020	0.514 ± 0.029	3.358	0.067	0.343
**AMY**	0.314 ± 0.029	0.372 ± 0.042	1.297	0.255	0.276	**DH**	**0.624** ± 0.059	**0.325** ± 0.051	**15.759**	**0.001** [1]	**1.154**
**DH**	**0.609** ± 0.040	**0.090** ± 0.053	**69.010**	**0.001** [1]	**1.731**	**VH**	0.478 ± 0.077	0.492 ± 0.055	0.023	0.880	0.054
**VH**	**0.576** ± 0.073	**0.287** ± 0.084	**7.597**	**0.006** [1]	**1.059**	**SUB**	0.260 ± 0.070	0.274 ± 0.053	0.036	0.849	0.053
**SUB**	0.226 ± 0.060	0.079 ± 0.069	2.808	0.094	0.575	**PHC**	**0.450** ± 0.049	**0.229** ± 0.051	**10.129**	**0.001** [1]	0.666
**PHC**	0.155 ± 0.046	0.108 ± 0.048	0.523	0.470	0.205	**Between RSC and:**					
**Between TAL and:**						**TAL**	0.523 ± 0.049	0.508 ± 0.049	0.047	0.828	0.058
**AMY**	**0.527** ± 0.027	**0.676** ± 0.018	**21.973**	**0.001** [2]	**0.753**	**AMY**	**0.356** ± 0.047	**0.559** ± 0.032	**12.963**	**0.001** [2]	**0.645**

**DH**	0.825 ± 0.055	0.699 ± 0.050	3.072	0.080	0.553	**DH**	0.571 ± 0.061	0.499 ± 0.060	0.757	0.384	0.308
**VH**	0.646 ± 0.040	0.606 ± 0.041	0.504	0.478	0.232	**VH**	0.542 ± 0.057	0.654 ± 0.069	1.698	0.193	0.455
**SUB**	0.425 ± 0.045	0.300 ± 0.056	3.180	0.075	0.445	**SUB**	0.516 ± 0.057	0.536 ± 0.058	0.84	0.772	0.081
**PHC**	**0.580** ± 0.038	**0.360** ± 0.043	**15.065**	**0.001** [1]	**0.733**	**PHC**	0.470 ± 0.052	0.485 ± 0.043	0.056	0.813	0.053
**Between AMY and:**						**Between DH and:**					
**DH**	**0.360** ± 0.032	**0.480** ± 0.025	**9.020**	**0.003** [2]	**0.866**	**VH**	**0.755** ± 0.036	**0.520** ± 0.059	**9.841**	**0.002** [1]	**1.156**
**VH**	**0.332** ± 0.043	**0.720** ± 0.027	**60.869**	**0.001** [2]	**1.375**	**SUB**	0.507 ± 0.059	0.386 ± 0.091	1.317	0.251	0.403
**SUB**	0.315 ± 0.043	0.349 ± 0.027	0.362	0.548	0.114	**PHC**	0.550 ± 0.049	0.483 ± 0.064	0.718	0.397	0.240
**PHC**	**0.540** ± 0.021	**0.380** ± 0.021	**23.179**	**0.001** [1]	**0.680**	**Between VH and:**					
**Between SUB and:**						**SUB**	0.537 ± 0.055	0.511 ± 0.072	0.083	0.774	0.103
**PHC**	0.429 ± 0.049	0.495 ± 0.043	1.069	0.301	0.228	**PHC**	0.534 ± 0.056	0.434 ± 0.040	2.200	0.138	0.414

Supplementary Table S1 - Mean correlation coefficient (Pearson's r) within each anatomical group; between one anatomical group and the remaining ones or between pairs of anatomical groups in CFC and CFC-5s groups. Generalized Linear Models; **[1]** Indicates $p < 0.050$, with higher absolute mean correlation coefficient in CFC group; **[2]** indicates $p < 0.050$, with higher absolute mean correlation coefficient in CFC-5s group. Standardized betas (β) were used as a measure of effect size. **AMY**: amygdala nuclei; **DH:** dorsal hippocampus; **FC:** frontal cortex; **mPFC:** medial prefrontal cortex; **PHC:** parahippocampal cortex; **RSC**: retrosplenial cortex; **SUB:** subicular complex; **TAL:** thalamic nuclei. **VH:** ventral hippocampus (major brain subdivisions). **CFC-5s:** contextual fear conditioning with 5-second interval; **CFC:** contextual fear conditioning.

Supplementary Table S2 – Overview of the CFC-5s and CFC functional networks

Measure	CFC p < 0.05	CFC-5s p < 0.05	GZLM Wald	GZLM p-value	K-S p-value	CFC p < 0.01	CFC-5s p < 0.01	GZLM Wald	GZLM p-value	K-S p-value
**Nodes**	49	49	NA	NA	NA	49	46	NA	NA	NA
**Edges**	946	930	NA	NA	NA	562	484	NA	NA	NA
**Connected Components**	1	1	NA	NA	NA	1	2	NA	NA	NA
**Graph Density**	0.804	0.790	NA	NA	NA	0.479	0.469	NA	NA	NA
**Network Diameter**	4	5	NA	NA	NA	5	5	NA	NA	NA
**Average Path Length**	1.678	1.755	NA	NA	NA	2.222	2.126	NA	NA	NA
**Modularity**	0.270	0.216	NA	NA	NA	0.480	0.324	NA	NA	NA
**Average Degree**	38.612 ± 2.157	37.918 ± 2.605	0.043	0.836	0.856	22.980 ± 1.538	21.087 ± 1.824	0.649	0.421	0.559
			23.533	**0.001** [2]	**0.001** [2]			35.542	**0.001** [2]	**0.001** [2]
**Average Weighted Degree**	29.616 ± 1.709	28.27 ± 2.061	0.258	0.612	0.699	19.220 ± 1.323	17.601 ± 1.544	0.653	0.419	0.559
			14.496	**0.001** [2]	**0.001** [2]			23.620	**0.001** [2]	**0.001** [2]
**Global Efficiency**	0.716 ± 0.005	0.711 ± 0.010	0.190	0.663	**0.037** [1]	0.574 ± 0.008	0.605 ± 0.010	2.834	0.092	**0.001** [1]
			47.208	0.001 [2]	**0.001** [2]			165.556	**0.001** [2]	**0.001** [2]
**Nodal Efficiency**	0.691 ± 0.014	0.680 ± 0.017	0.235	0.628	0.856	0.554 ± 0.014	0.574 ± 0.017	0.803	0.370	0.142
			17.308	**0.001** [2]	**0.001** [2]			45.439	**0.001** [2]	**0.001** [2]
**Average Cluster Coefficient**	0.702 ± 0.013	0.695 ± 0.024	0.318	0.573	0.961	0.673 ± 0.019	0.650 ± 0.034	2.334	0.127	0.473
			4.854	**0.028** [2]	**0.001** [2]			4.583	**0.032** [2]	**0.020** [2]

Supplementary Table S2 – CFC and CFC-5s functional networks had similar topological measures. **Nodes:** number of brain regions; **edges:** number of correlation coefficients above the threshold level, counted in both directions; **connected components:** number of subgraphs in which all pairs of nodes are connected with each other via a path; **graph density:** how many edges the network has, from the total of possible ones (a complete network would have graph density of 1); **network diameter:** the longest distance (sum of edges) between any two nodes; **average path length:** the network average distance (number of edges) between all pair of nodes (Watts and Strogatz, 1998); **modularity:** the quality of the partition of nodes into communities, ranging from -1 to +1 (Blondel et al., 2008); **average degree:** the average number of edges (functional connections) per node (brain region); **average weighted degree:** the average degree pondered by the weight of the correlation coefficient; **global efficiency:** the network average of

the nodal efficiencies of all nodes (computed for each node as the inverse of the harmonic mean of the shortest path length, the minimum number of edges, between the node and all the others). Includes disconnected nodes (Latora and Marchiori, 2001); **local efficiency:** the network average of the nodal efficiencies of the neighbors of a node, excluding the node itself (Latora and Marchiori, 2001); **nodal efficiency:** the network average of the harmonic mean of the shortest path length (minimum number of edges) between the node and all the other nodes in the network (Latora and Marchiori, 2001); **average cluster coefficient**: the network average of the number of neighbors connected, from the total number of possible functional connections among the neighbors of a node (i.e., number of connected triangles from the possible ones; Watts and Strogatz, 1998); Data are shown in mean ± standard error. Generalized Linear Models (**GZLM**) or two-sample Kolmogorov-Smirnov (**K-S**) test. **1** indicates $p < 0.050$ in the between-groups comparisons (CFC x CFC-5s). **2** indicates $p < 0.050$ in the within-groups comparisons ($p < 0.05$ x $p < 0.01$). **NA:** GZLM or K-S tests are not applicable.

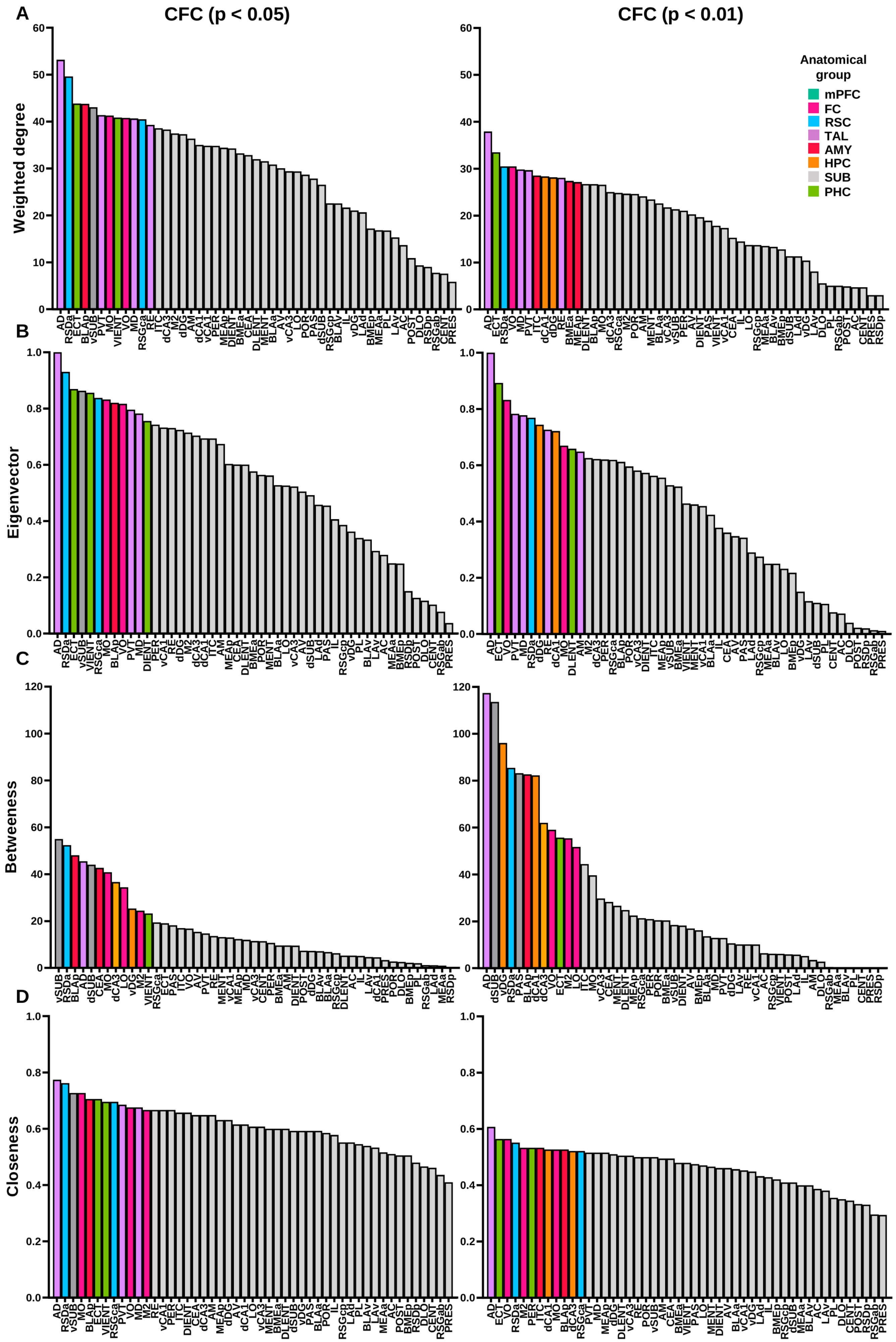

A
CFC (p < 0.05)
CFC (p < 0.01)
Weighted degree
Anatomical group
mPFC
FC
RSC
TAL
AMY
HPC
SUB
PHC
B
Eigenvector
C
Betweeneness
D
Closeness

Supplementary Figure S2 - Brain regions ranked by their centrality measures in the CFC functional networks. Centrality measures of weighted degree **(A)**, eigenvector **(B)**, betweenness **(C)** and closeness **(D)** in the CFC networks using $p < 0.05$ to select the significant coefficient correlations (left) or $p < 0.01$ (right). Colored bars represent the upper 25% of the brain regions, considered high centrality nodes. Colors reflect the anatomical group to which the brain region belongs (scale, right). We described the full name of the brain regions in Table 1. **CFC:** contextual fear conditioning.

Supplementary Table S3 – Comparison of centrality measures between the CFC-5s and CFC network

	Weighted degree		Eigenvector		Betweenness		Closeness	
**Region**	**$p < 0.05$**	**$p < 0.01$**	**$p < 0.05$**	**$p < 0.01$**	**$p < 0.05$**	**$p < 0.01$**	**$p < 0.05$**	**$p < 0.01$**
**AC**	0.529	0.667	0.412	0.778	**0.001** [2]	0.333	0.471	0.556
**AD**	0.350	0.400	0.700	0.500	0.900	0.200	0.600	0.900
**AM**	0.100	0.350	0.050	0.100	0.400	0.750	0.650	0.550
**AV**	1.000	1.000	0.600	0.611	1.000	0.778	1.000	0.833
**BLAa**	0.200	0.300	**0.001** [2]	**0.050** [2]	0.550	0.150	0.300	**0.050** [2]
**BLAp**	0.700	0.526	0.550	0.474	0.400	0.211	0.800	0.526
**BLAv**	0.200	0.200	**0.050** [2]	**0.050** [2]	0.900	0.550	0.300	0.200
**BMAa**	0.250	0.526	0.150	0.316	0.650	0.579	0.350	0.316
**BMAp**	**0.050** [2]	0.100	**0.050** [2]	0.200	0.400	0.300	0.100	0.200
**CEA**	0.150	0.300	0.250	0.350	0.250	0.550	0.250	0.600
**dCA1**	0.300	1.000	0.650	0.700	0.750	0.400	0.800	1.000
**dCA3**	0.650	0.450	0.800	0.550	0.450	0.300	0.650	0.650
**dDG**	0.300	0.100	0.600	0.100	0.600	0.400	0.550	0.300
**dSUB**	0.550	0.900	0.900	0.500	**0.001** [1]	0.700	0.550	0.950
**DIENT**	0.100	0.474	**0.050** [2]	0.316	0.600	0.947	**0.050** [1]	0.526
**DLENT**	0.800	0.778	0.300	0.778	0.850	0.778	0.350	0.778
**DLO**	0.200	0.579	**0.050** [2]	0.526	0.400	0.053	0.250	0.368
**ECT**	0.750	0.150	0.600	0.300	0.700	0.400	0.500	0.400
**IL**	**0.050** [2]	0.750	**0.001** [2]	0.900	0.500	0.900	0.350	1.000
**ITC**	0.550	0.350	0.400	0.900	0.800	0.150	0.850	0.850
**LAd**	0.900	0.150	0.400	0.100	0.850	0.500	0.850	0.100
**LAv**	0.300	**0.050** [2]	0.250	**0.001** [2]	0.450	0.150	0.150	**0.001** [2]
**LO**	0.500	0.526	0.600	0.579	0.500	0.105	0.700	0.579
**MD**	0.100	0.850	0.100	0.550	0.450	**0.001** [2]	0.050	0.450
**MEAa**	0.800	0.650	0.800	0.500	0.150	0.500	0.750	0.450
**MEAp**	0.700	**0.050** [1]	0.700	0.300	0.200	0.500	0.650	0.450
**MENT**	0.150	0.100	0.300	0.300	0.650	0.500	0.500	0.550
**MO**	0.500	0.800	0.750	0.950	0.500	0.450	0.450	1.000
**PAS**	**0.050** [1]	0.211	**0.001** [1]	0.158	0.400	0.053	**0.001** [1]	0.263
**PER**	0.250	0.750	0.100	0.550	**0.050** [1]	0.500	0.200	0.800
**PL**	0.278	0.692	0.222	0.769	0.600	0.846	0.611	0.538
**POR**	**0.001** [1]	**0.001** [1]	**0.001** [1]	0.111	0.800	0.444	**0.050** [1]	0.222
**POST**	0.550	0.526	0.650	0.895	0.700	0.684	0.850	0.053
**PVT**	0.550	**0.050** [1]	1.000	**0.050** [1]	1.000	0.700	0.500	0.650
**RE**	0.950	0.800	0.750	0.850	0.200	0.350	0.700	0.700
**RSDa**	**0.001** [1]	0.300	**0.001** [1]	0.200	0.550	0.200	0.150	0.550
**RSDp**	0.053	0.158	**0.001** [2]	0.105	0.158	0.684	0.158	0.211
**RSGab**	0.150	0.650	0.150	0.850	0.300	0.750	0.200	**0.050** [2]
**RSGca**	1.000	0.850	0.800	0.750	1.000	0.500	0.600	0.450
**RSGcp**	0.350	0.895	0.200	0.737	0.750	0.842	0.350	0.684
**vCA1**	**0.001** [2]	0.900	**0.001** [2]	0.950	0.600	0.850	**0.001** [2]	0.550
**vCA3**	0.750	0.600	0.950	0.650	0.650	0.900	0.850	0.550
**vDG**	0.250	0.263	0.100	0.105	0.650	0.105	0.500	0.526
**VIENT**	**0.050** [1]	0.053	**0.050** [1]	0.053	0.750	0.789	**0.050** [1]	0.316
**vSUB**	0.400	0.400	0.350	0.450	0.350	0.550	0.300	0.650
**VO**	0.700	1.000	0.800	0.750	0.250	1.000	0.750	1.000

Supplementary Table S3 – Comparison of centrality measures between the CFC-5s and CFC networks. We compared each brain region in each one of the four centrality measures (weighted degree, eigenvector, betweenness, and closeness), and each threshold level ($p < 0.050$ and $p < 0.010$) **[1]** Indicates higher centrality in the CFC and **[2]** in the CFC-5s functional network. We described the full name of the brain regions in Table 1.
